# Structure–Activity Relationship of the Thiacalix[4]arenes Family with Sulfobetaine Fragments: Self-Assembly and Cytotoxic Effect against Cancer Cell Lines

**DOI:** 10.3390/molecules27041364

**Published:** 2022-02-17

**Authors:** Luidmila Yakimova, Aisylu Kunafina, Aigul Nugmanova, Pavel Padnya, Alexandra Voloshina, Konstantin Petrov, Ivan Stoikov

**Affiliations:** 1 A.M. Butlerov’ Chemistry Institute, Kazan Federal University, 18 Kremlevskaya Street, 420008 Kazan, Russia; ais.kunaf@yandex.ru (A.K.); aygul9pul9@mail.ru (A.N.); padnya.ksu@gmail.com (P.P.); 2 Arbuzov Institute of Organic and Physical Chemistry, FRC Kazan Scientific Center of RAS, 8 Arbuzov Street, 420088 Kazan, Russia; microbi@iopc.ru (A.V.); kpetrov2005@mail.ru (K.P.)

**Keywords:** sulfobetaines, thiacalixarene, self-assembly, silver ions, cytotoxicity, cancer cell lines

## Abstract

Regulating the structure of macrocyclic host molecules and supramolecular assemblies is crucial because the structure–activity relationship often plays a role in governing the properties of these systems. Herein, we propose and develop an approach to the synthesis of the family of sulfobetaine functionalized thiacalix[4]arenes with regulation of the self-assembly and cytotoxic effect against cancer cell lines. The dynamic light scattering method showed that the synthesized macrocycles in *cone*, *partial cone* and *1,3-alternate* conformations form submicron-sized particles with Ag^+^ in water, but the particle size and polydispersity of the systems studied depend on the macrocycle conformation. Based on the results obtained by ^1^H and ^1^H-^1^H NOESY NMR spectroscopy and transmission electron microscopy for the macrocycles and their aggregates with Ag^+^, a coordination scheme for the Ag^+^ and different conformations of *p*-*tert*-butylthiacalix[4]arene functionalized with sulfobetaine fragments was proposed. The type of coordination determines the different shapes of the associates. Cytotoxic properties are shown to be controlled by the shape of associates, with the highest activity demonstrated by thiacalix[4]arenes in *partial* *cone* conformation. This complex *partial cone*/Ag^+^ is two times higher than the reference drug imatinib mesylate. High selectivity against cervical carcinoma cell line indicates the prospect of their using as components of new anticancer system.

## 1. Introduction

Multiple drug resistance (MDR) is a growing public health concern worldwide [1,2,3,4,5]. The main challenge faced by many researchers is the resistance to cancer and various bacterial and viral infections. It leads to the ineffectiveness of therapy. It is now well established that inappropriate use of antimicrobial, antiviral, anticancer agents or use of ineffective dosage forms [6], as well as premature termination of treatment can lead to the development of drug resistances [7]. MDR is not limited to bacterial infections; this phenomenon is also characteristic of mycoses. Interestingly, cancer cells are drug resistant too [8,9]. Moreover, cancer cells can be compared to superbugs since almost 50% of cancer cells do not respond to any anticancer drugs [10]. Existing research recognizes that any drug effects both on cancer and on healthy cells [11].

The clinical development portfolio of new antimicrobial drugs is extremely small. In 2019, the World Health Organization (WHO) identified 32 antibiotics that are in clinical development and are intended to treat pathogens included in the WHO list of priority pathogens. Only six antibiotics are classified as innovative [12]. In addition, the lack of availability of quality antimicrobial drugs remains a major problem.

Several approaches to solving the problem of MRD have been proposed. One of the most widely used approaches is the use of innovative delivery systems (liposomes, nanofibers, lipid particles, etc.) for the encapsulation (solubilization) of drugs existing on the world market. The bac/virus resistance issue will be solved by developing new methods for the delivery of existing drugs rather than by synthesizing new medicines. Thus, the effect of several antibiotics, encapsulated directly in nanofibers, was tested on laboratory cultures of various microbes [13]. Antibiotics have proven extremely effective against a variety of pathogenic bacteria and fungi, including *Escherichia coli* and *Pseudomonas aeruginosa*, two of the most drug-resistant organisms. The packaging of antibacterial substances in dosage forms makes the action of drugs more targeted. New dosage forms remain effective for a longer period than with the usual method of their delivery.

The second approach is the development of nanostructures without the participation of a drug through the laws and approaches of supramolecular chemistry [14]. This direction can become the basis for the creation of a new class of anticancer systems (“nano anticancer drug”) with controlled activity, selectivity, and biological compatibility. Among inorganic nanoparticles, both Ag nanoparticles [15] and Ag^+^ [16,17] cations are the most widely used, for which a pronounced ability to inactivate viruses, smallpox and influenza A-1, B has been shown [18,19]. They are active against some enteroviruses, adenoviruses and human immunodeficiency virus. Moreover, it has been shown that the toxicity of both Ag^+^ ions and Ag nanoparticles is quite high [20]. Ag nanoparticles can be toxic due to their small size; on the other hand, Ag nanoparticles can be toxic because they release Ag^+^, which are well known for their antibacterial and destructive effects on cell membranes [21,22]. Therefore, the regulation of the cytotoxic effect of Ag^+^ on normal and tumor cell cultures is undoubtedly a non-trivial task.

In this regard, the development of new approaches of supramolecular chemistry and the identification of the structure–activity relationship is an important and promising problem [23,24]. Macrocyclic platforms—thiacalix[4]arenes, can be used for the synthesis of compounds with a regulated ability to self-assembly [25,26,27]. Due to the ability to fix the macrocyclic ring in several conformations and the relative ease of its functionalization, functional groups can be arranged in a predetermined manner in space. It determines the tendency to self-assembly into nanostructures under the influence of the substrate (ions, molecules) [28,29,30].

In this work, we synthesize sulfobetaine derivatives of thiacalix[4]arene in three conformations using the “template” synthesis method (with alkali metal ions as a template) to create multivalent structures. In the presence of Ag^+^, these derivatives are capable to self-assemble into associates with different architectures (shapes) and, as a consequence, with cytotoxic selectivity for the cervical carcinoma cell line.

## 2. Results and Discussion

### 2.1. Synthesis of Compounds ***3**–**5***

**Scheme 1:**
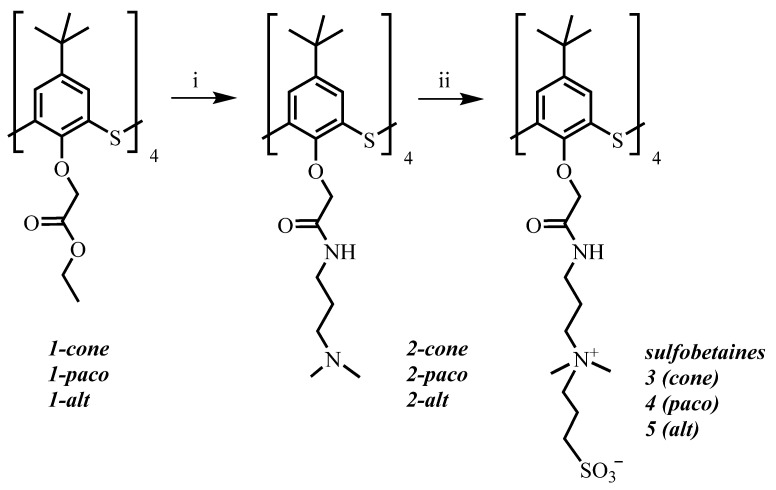
Reagents and conditions: **i**—*N*,*N*-dimethylpropane-1,3-diamine, C_6_H_5_CH_3_/CH_3_OH, reflux; **ii**—1,3-propanesultone, CH_3_CN, reflux.

In our previous work, we synthesized derivatives of *p-tert*-butylthiacalix[4]arene with sulfobetaine moieties on the lower rim in *cone* conformation and studied their capability of self-assembling in the presence of Ag (I) [31]. Due to the presence of sulfide bridges in the thiacalix[4]arene, the conformational mobility of the macrocyclic ring increases, which facilitates the preparation of two additional conformers (*partial cone* and *1,3-alternate*) [32]. In this case, the arrangement of substituents at phenolic oxygen in space is different for all stereoisomers, which makes it possible to organize an orientation of the binding sites that is individual for each conformer, and this will undoubtedly lead to selectivity of interaction with the substrate. In this regard, in order to evaluate the effect of the conformation of the macrocyclic platform on self-assembly with Ag (I) cations, we synthesized three conformers (*cone*, *partial cone* and *1,3-alternate*) of thiacalix[4]arene containing four sulfobetaine fragments (**3**–**5**) (Scheme 1). Aminolysis of *p-tert*-butylthiacalix[4]arenes tetrasubstituted at the lower rim by ester fragments in *cone* (**1***-cone*), *partial cone* (**1***-paco*) and *1,3-alternate* (**1***-alt*) conformation by *N*,*N*-dimethylpropane-1,3-diamine give the tetrasubstituted thiacalix[4]arene derivatives containing tertiary amino groups (**2***-cone**,*** **2***-paco**,*** **2***-alt*) [33]—the precursors for the synthesis of sulfobetaine macrocyclic compounds.

Next, the reactions of compounds **2** in *cone*, *partial cone* and *1,3-alternate* conformations in acetonitrile with 1,3-propanesultone were attempted for 72 h. The reactions with 1,3-propanesultone gave products **3**–**5** in a yields of 79, 95 and 91%. The complete set of ^1^H, ^13^C NMR and IR spectroscopy and ESI mass spectrometry data confirm the formation of the tetrasubstituted products **3**–**5** (Figures S1–S12, see details in Supplementary Materials).

### 2.2. Self-Assembly of Water-Soluble Sulfobetaines ***3**–**5***

With the sulfobetaine macrocycles **3**–**5** in hand, we investigated their aggregation in water (Figures S13–S42, ESI). Colloidal solutions of macrocycles **3**–**5** were characterized by a high value of dispersity (PDI = 0.31–0.91) and particle size from 317 to 421 nm (Tables S1–S3). In the range of concentrations studied for sulfobetaine **3** in *cone* conformation, the aggregate size decreases (from 421 to 317 nm) with a lowering concentration (1 × 10^−4^ − 1 × 10^−6^ M), while the dispersity also decreases (Table S1). After the addition of AgNO_3_ at a 1:1 molar ratio (**3**:Ag^+^), nanosized particles with a hydrodynamic diameter of about 168 nm and a low dispersity (PDI = 0.17) were formed. At the same time, with a decrease in the concentration of **3**, an opposite tendency is observed (Table S1). Obviously, with a higher macrocycle concentration, some charge compensation occurs, which causes higher monodispersity.

Sulfobetaine **5** in *1,3-alternate* conformation in all systems, at various concentrations of Ag (I), was not capable of forming stable monodisperse systems (Table S3). In the concentration range studied (1 × 10^−4^ – 1 × 10^−6^ M), there is no definite trend in the size change, while the dispersity increases (from 0.38 to 0.91) with a lowering concentration (Table S3). The addition of Ag (I) to the solution of **5** in stoichiometric ratios 1:1, 1:4, 1:10 did not leаd to the formation of a system with a monomodal distribution. Therefore, both the aggregate size formed by macrocycle **5** and their dispersity weakly depend on the concentration.

A more complicated picture was observed for sulfobetaine **4** in *partial cone* conformation. In the concentration studied (1 × 10^−4^ M), macrocycle **4** forms monodisperse systems at all the ratios studied with Ag (I) with a size of 123–187 nm (Table S2). Dilution of the system to 1 × 10^−5^ M led to a violation of the stability of the system and, as a consequence, to an increase in the polydispersity and size of the resulting aggregates. At a lower concentration (1 × 10^−6^ M), no associates were found.

Note that Ag (I), rather than the reduced form of Ag (0), participates in the formation of aggregates with macrocycles **3**–**5**. This is confirmed by the UV spectra of the studied systems, which were recorded after 1 day, 3 days, and 7 days. The spectra lack the absorption band of reduced silver at 430 nm (Figures S43–S45).

**Figure 1:**
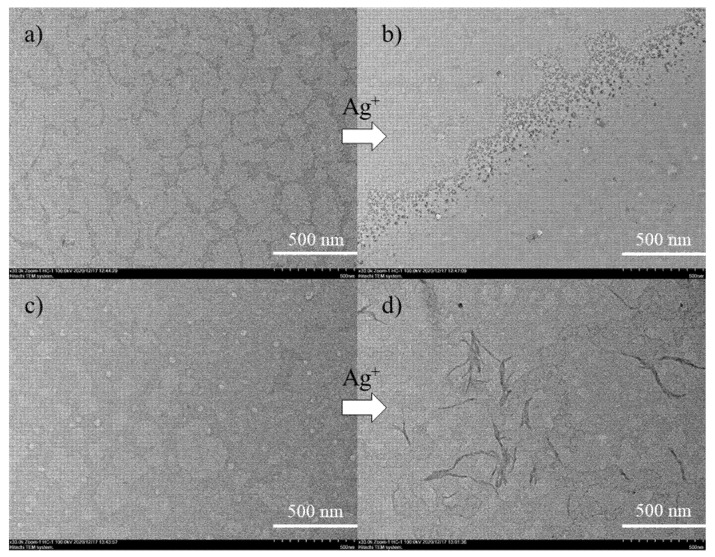
TEM images of (**a**) **3**, (**b**) **3**/Ag^+^, (**c**) **4** and (**d**) **4**/Ag^+^ associates (1:4 molar ratio) in water (1 × 10^−4^ M).

The formation of associates in the presence of Ag^+^ was also recorded in the solid phase by TEM (Figure 1). The shape of the associates formed by macrocycles **3** and **4** is different. In the case of associates **3**/Ag^+^, spherical particles are formed, while the association of macrocycle **4** with Ag^+^ gives associates of an extended shape.

**Figure 2:**
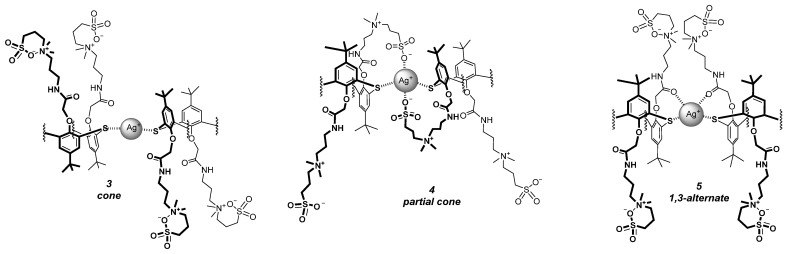
Coordination of the metals by the isomers of thiacalix[4]arenes **3**–**5** proposed for various conformations.

Obviously, such a difference in the shape of associates is due to the structure of the macrocycle, namely, to the different arrangement of substituents relative to the macrocyclic cavity (*cone*, *partial cone* and *1,3-alternate*). To confirm our assumption, we studied the systems of macrocycles **3**–**5** and their associates with Ag^+^ by ^1^H NMR spectroscopy (Figure 2, Figures S46 and S47).

Unfortunately, no significant changes in the ^1^H NMR spectrum for the **3**/Ag^+^ system were found (Figure S46). We used the 2D 1H,1H-nuclear Overhauser effect spectroscopy (NOESY) NMR method (Figures S48 and S49) to confirm the formation and structure of associates. There are observed cross-peak between the methylene protons of CH_2_SO_3_^−^ and the methyl protons of Me_2_N^+^ of sulfobetaine fragment 1H,1H-NOESY NMR spectrum. The presence of these cross peaks indicates a spatially close location of CH_2_SO_3_^−^ and the methyl protons of Me_2_N^+^ in sulfobetaine fragments of pillar[5]arenes **3**. Due to electrostatic interactions, the charge in the sulfobetaine part is compensated, and a stable six-membered ring is formed (Figure 2). The coordination of Ag^+^ is possible with bridging sulfur atoms and at the oxygen atoms of the carbonyl groups of the receptor [34,35]. Apparently, such an organization of the sulfobetaine fragment in the symmetric *cone* conformation in space and the coordination of Ag^+^ over bridging sulfur atoms does not lead to significant shielding of the macrocycle fragments. This is not reflected in the ^1^H NMR spectrum of the **3**/Ag^+^ associates.

In the ^1^H NMR spectrum of **5**/Ag^+^ associates formed by a macrocycle in the *1,3-alternate* conformation, significant changes were found only in the region of oxymethylene protons. The signal shifts to the low-field region by 0.16 ppm (Figure S47). At the same time, in the ^1^H–^1^H NOESY NMR spectrum of macrocycle **5**, a cross-peak between the methylene group of CH_2_SO_3_^−^ and the methyl groups of Me_2_N^+^ is also observed (Figure S52), which indicates the formation of a cyclic structure between the charged groups of the sulfobetaine fragment. In this case, the Ag^+^ is so oriented in the space of the symmetric conformation of the macrocycle that it causes shielding of only oxymethylene fragments of OCH_2_ and does not promote self-assembly into ordered aggregates of the same size (Figure S47). This is possible only in the case of a similar arrangement of the Ag^+^ cation, as shown in the Figure 2.

**Figure 3:**
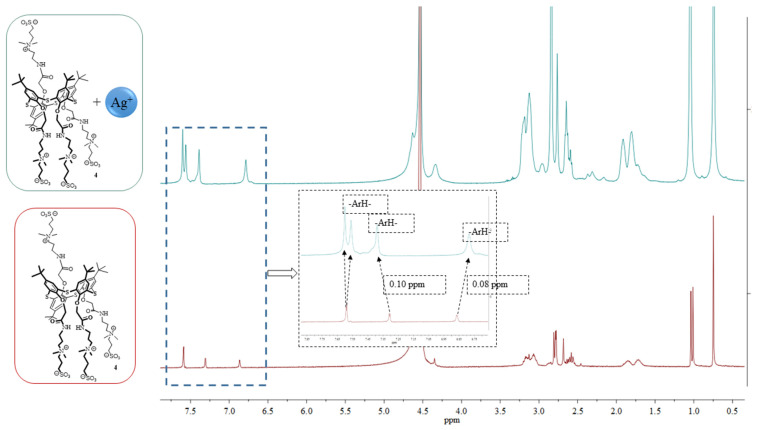
^1^Н NMR spectra of macrocycle **4** (1 × 10^−3^ M) and associates **4**/Ag^+^ at a ratio of 1:4 in D_2_O at 298 K.

The most significant changes in the ^1^H NMR spectrum are observed for the **4**/Ag^+^ system (Figure 3). Macrocycle **4** in *paco* conformation is more dissymmetric than macrocycles in *cone* and *1,3-alternate* conformations and is more conformationally labile. Proton signal shifts are observed for almost all fragments, except for the *tert*-butyl groups of the aromatic ring and methyl groups at the quaternized nitrogen atom. The greatest changes were observed for aromatic protons, as well as methylene protons of sulfobetaine fragments.

**Figure 4:**
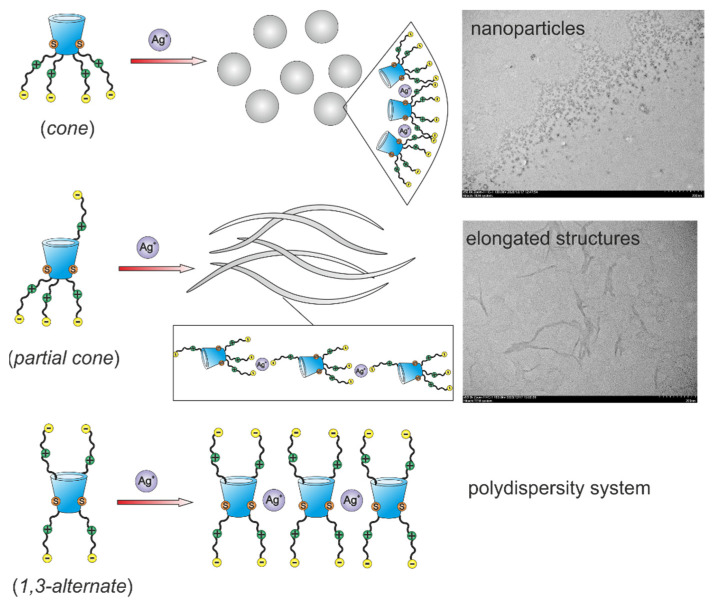
The possible structures of *p-tert*-butyl thiacalix[4]arenes **3**–**5** aggregated with Ag^+^ cations.

It should be noted that in the ^1^H–^1^H NOESY NMR spectrum of macrocycle **4**, in contrast to macrocycles **3** and **5**, there are no cross peaks between the methylene group of CH_2_SO_3_^−^ and the methyl groups of Me_2_N^+^ (Figure S50). This means that the conformational lability of macrocycle **4** increases the mobility of the fragments relative to the macrocyclic ring. This complicates such an orientation of groups in space, which would lead to intermolecular cyclization of the sulfobetaine fragment. As a result, coordination of the Ag^+^ cation becomes possible not only at bridging sulfur atoms and oxygen atoms of carbonyl groups but also through electrostatic interactions with the sulfo group of the betaine fragment (Figure 2). Such electrostatic interactions “glue” the thiacalix[4]arene molecules into extended structures, which we observe in the images obtained by the method of transmission electron spectroscopy (Figure 1). The coordination of Ag^+^ for different conformations of *p-tert*-butylthiacalix[4]arene functionalized with sulfobetaine fragments is schematically shown in Figure 4.

### 2.3. Cytotoxicity of Test Compounds ***3**–**5*** on Cancer and Non-Cancer Human Cell Lines

It is known that Ag nanoparticles are highly cytotoxic [20]. It is worth noting that Ag nanoparticles can be toxic because they release Ag^+^, which are well known for their antibacterial and other destructive behavior [36]. Moreover, nanoparticles enhance the effect of ions [37,38]. Taking these data into account, the cytotoxicity of the synthesized nanostructures based on sulfobetaine thiacalix[4]arenes and Ag^+^, as the most aggressive form of silver, was evaluated. We studied the cytotoxic effect of thiacalix[4]arene derivatives **3**–**5** and their aggregates **3**/Ag^+^ and **4/**Ag^+^ on cancer human cell lines MCF-7, M-HeLa, HuTu 80 and on non-cancer cells (change liver) in the concentration range of 1–100 µM. This concentration range is recommended for screening for new anticancer agents (1–100 µМ) [39].

It was shown that compounds **3**–**5** do not demonstrate cytotoxic activity against all cell lines (Table 1). It can be seen that among the tested compounds, the **4**/Ag^+^ aggregate selectively decreases the viability of cervical carcinoma cells (M-HeLa). In terms of cytotoxic activity, this complex is twice as great as the reference anti-cancer drug imatinib mesylate. Its cytotoxicity against Chang liver cells differs slightly from the known anti-cancer drug. It should be noted that all studied compounds and **3**/Ag^+^ aggregates were inactive against MCF-7 and HuTu 80 to cell lines.

The selectivity of compounds for cancer cells is an important criterion for assessing the cytotoxic effect. For this purpose, the selectivity index (SI) was calculated as the ratio between the IC_50_ value for non-cancer cells and the IC_50_ for cancer cells. Compounds with SI ≥ 3 are usually selective [36]. The SI values for lead system **4**/Ag^+^ on the M-HeLa cell line were 2.2. The reference drug Imatinib mesylate was significantly inferior to the leading compounds in selectivity.

The cytotoxic selectivity of **4**/Ag^+^ aggregates may be due to their architecture. It is known from the literature that the shape of nanostructures can have a critical effect on the effectiveness of the cytotoxic action [37]. Indeed, the association of macrocycle **4** in the presence of Ag^+^ leads to the formation of extended nanostructures, while the association of macrocycle **3** with the addition of AgNO_3_ gives spherical aggregates (Figure 1).

## 3. Materials and Methods

### 3.1. General

All NMR experiments were performed on the Bruker Avance-400 spectrometer. Chemical shifts were reported relative to deuterated water as an internal standard. Attenuated total internal reflectance, FTIR spectra of all compounds have been recorded using Spectrum 400 (Perkin Elmer) 4000 to 400 cm^−1^ range. Analysis of elements C, H and N have been recorded using a Perkin-Elmer 2400 CHN elemental analyzer. MS experiments (electrospray ionization) were carried out using a AmazonX spectrometer (Bruker Daltonics, Bremen, Germany). The ionization parameters were as follows: positive ion mode; capillary voltage 4.5 kV, drying gas of 10 L/min nitrogen at 300 °C. Mass analyzer scanned from 100 to 2800 u. Melting points were determined using the Boetius Block apparatus. Chemicals were purchased from Sigma-Aldrich.

Compounds **1**, **2** were synthesized by the authors of [40,41].

### 3.2. General Procedure for the Synthesis of Compounds ***3**–**5***

The compounds **2** (**2**-*cone*, **2**-*paco*, **2**-*alt*) (0.20 g, 0.16 mmol) and 1,3-propanesultone (0.64 mmol) was mixed in 15 mL of acetonitrile. The reaction mixture was refluxed for 72 h. After that, solvent was removed under reduced pressure and the residue was dried in vacuo.

5,11,17,23-Tetra-*tert*-butyl-25,26,27,28-tetrakis[(N-(3′,3′-dimethyl-3′-{3″-sulfonatopropyl})ammoniumpropyl)-carbamoylmethoxy]-2,8,14,20-tetrathiacalix[4]arene (*cone*) (**3**) was synthesized in our research group early [32]. Spectral data are presented in Supporting Information (Figures S1–S4).

5,11,17,23-Tetra-*tert*-butyl-25,26,27,28-tetrakis[(N-(3′,3′-dimethyl-3′-{3″-sulfonatopropyl})ammoniumpropyl)-carbamoylmethoxy]-2,8,14,20-tetrathiacalix[4]arene (*partial cone*) (**4**).

Yield 0.26 g (95%). ^1^H NMR (DMSO-*d_6_*, δ, ppm, *J*/Hz): 1.01 (s, 18H, (CH_3_)_3_C), 1.28 (s, 9H, (CH_3_)_3_C), 1.29 (s, 9H, (CH_3_)_3_C), 1.80–2.04 (m, 16Н, -N^+^CH_2_CH_2_CH_2_SO_3_^-^, -NCH_2_CH_2_CH_2_NH), 2.45 (m, 8Н, -N^+^CH_2_CH_2_CH_2_SO_3_^−^), 2.97–3.03 (m, 24H, (CH_3_)_2_N^+^), 3.14–3.30 (m, 16H, N^+^CH_2_CH_2_CH_2_NH, -N^+^CH_2_CH_2_CH_2_SO_3_^−^), 3.4 (m, 8H, N^+^CH_2_CH_2_CH_2_NH), 4.36 (d, 2H, OCH_2_CO, ^2^*J*_HH_ = 13.6 Hz), 4.46 (s, 2H, OCH_2_CO), 4.54 (s, 2H, OCH_2_CO), 4.82 (d, 2H, OCH_2_CO, ^2^*J*_HH_ = 13.6 Hz), 7.02 (m, 2Н, ArH), 7.64 (m, 2Н, ArH), 7.67 (s, 2Н, ArH), 7.76 (s, 2Н, ArH), 8.32 (m, 2Н, CONH), 8.45 (m, 2Н, CONH). ^13^C NMR (DMSO-*d_6_*, δ, ppm): 169.21, 168.40, 167.51, 160.02, 157.70, 147.04, 146.08, 135.92, 135.31, 134.44, 128.48, 127.95, 127.49, 126.92, 73.17, 63.07, 62.67, 61.46, 61.35, 50.49, 48.09, 36.09, 34.45, 34.37, 34.28, 31.48, 31.19, 22.76, 22.68, 19.32. FTIR ATR (ν,cm^−1^): 1663 (С=О), 2954 (N^+^), 3429 (NH). El. Anal. Calcd for C_80_H_128_N_8_O_20_S_8_ (%): C 54.03%, H 7.25%, N 6.30%, S 14.42%. Found (%): C 54.47%, H 7.17%, N 6.57%, S 14.41%. MS (ESI): calculated 911.339 [M+2Na]^2+^, 615.222 [M+3Na]^3+^, 607.895 [M+2Na+H]^3+^, found: 911.337 [M+2Na]^2+^, 615.221 [M+3Na]^3+^, 607.893 [M+2Na+H]^3+^.

5,11,17,23-Tetra-*tert*-butyl-25,26,27,28-tetrakis[(N-(3′,3′-dimethyl-3′-{3″-sulfonatopropyl})ammoniumpropyl)-carbamoylmethoxy]-2,8,14,20-tetrathiacalix[4]arene (*1,3-alternate*) (**5**).

Yield 0.25 g (91%). ^1^H NMR (DMSO-*d_6_*, δ, ppm, *J*/Hz): 1.20 (s, 36H, (CH_3_)_3_C), 1.89 (m, 8Н, -NCH_2_CH_2_CH_2_NH), 1.99 (m, 8Н, -N^+^CH_2_CH_2_CH_2_SO_3_^−^), 2.06 (m, 8Н, -N^+^CH_2_CH_2_CH_2_SO_3_^−^), 3.01 (s, 24H, (CH_3_)_2_N^+^), 3.18 (m, 8Н, -N^+^CH_2_CH_2_CH_2_SO_3_^−^), 3.30 (m, 8H, NCH_2_CH_2_CH_2_NH), 3.43 (m, 8H, NCH_2_CH_2_CH_2_NH), 4.01 (s, 8H, OCH_2_CO), 7.60 (s, 8Н, ArH), 8.06 (m, 4Н, CONH). ^13^C NMR (DMSO-*d_6_*, δ, ppm): 167.79, 157.64, 146.54, 133.50, 127.98, 71.54, 62.84, 61.39, 50.52, 48.09, 39.90, 36.19, 34.37, 31.27, 22.84, 19.32. FTIR ATR (ν,cm^−1^): 1657 (С=О), 2957 (N^+^) 3300 (NH). El. Anal. Calcd for C_80_H_128_N_8_O_20_S_8_ (%): C 54.03%, H 7.25%, N 6.30%, S 14.42%. Found (%): C 54.36%, H 7.28%, N 6.47%, S 14.40%. MS (ESI): calculated 1777.7 [M+H]^+^, 889.4 [M+2H]^2+^, found: 1777.6 [M+H]^+^, 889.8 [M+2H]^2+^.

### 3.3. Transmission Electron Microscopy (TEM)

TEM images were obtained using a Hitachi HT7700 Exalens microscope, Japan. The images were acquired at an accelerating voltage of 100 keV. Samples were dispersed on 300 mesh copper grids with continuous carbonformvar support films.

### 3.4. Dynamic Light Scattering (DLS)

*Hydrodynamic Size of the Particles*. Zetasizer Nano ZS instrument (Malvern Instruments, Worcestershire, UK) determined the particle size at 293 K. The source of laser radiation was a He-Ne gas laser with a power of 4 mW power (λ_operating_ = 633 nm). The collected signals were treated in terms of frequency and phase analysis of scattered light using software attached to the device. All measurements were carried out at a 173° scattering angle. Deionized water with resistivity > 18.0 MΩ cm (Millipore-Q) was used for the preparation of the solutions. Synthesized *p-tert*-butylthiacalix[4]arenes **3**–**5** were dissolved in water at concentrations used in research (from 3 × 10^−6^ M to 3 × 10^−4^ M).

*Electrokinetic Potentials*. Electrokinetic (ζ) potentials were determined by electrophoretic light scattering on the Zetasizer Nano ZS (Malvern Instruments, Worcestershire, UK). Samples were prepared for the DLS measurements and transferred with the syringe to the disposable folded capillary cell for measurement. The ζ potentials were measured using the Malvern M3-PALS method and averaged from five measurements.

### 3.5. UV–Visible Spectroscopy

UV–visible spectra were recorded on the Shimadzu UV-3600 spectrophotometer at 293 K. Measurements were carried out in quartz cells with pathway lengths of 1.0 cm. AgNO_3_ were used as received. The 1 × 10^−4^ М solution of the AgNO_3_ (300 μL) in water was added to 0.3 mL of the solution of host (1 × 10^−4^ M) in water and diluted to final volume of 3 mL with water.

### 3.6. Сytotoxicity Assay

The M-HeLa clone 11 human, epithelioid cervical carcinoma, strain of HeLa, clone of M-Hela; human duodenal adenocarcinoma (HuTu 80); human breast adenocarcinoma cells (MCF-7) from the Institute of Cytology (Sankt-Petersburg) and human change liver from the collection and Research Institute of Virology, Russian Academy of Medical Sciences (Moscow) were used.

Cell viability was estimated using multifunctional Cytell Cell Imaging system (GE Health Care Life Science, Sweden) and Cell Viability Bio App. The cells were cultured in a standard Eagle’s nutrient medium (PanEco) and supplemented with 10% fetal calf serum and 1% nonessential amino acids. The cells were seeded in a concentration of 10^5^ cells/mL in 96-well plates (Eppendorf), then 150 μL of standard Eagle’s medium (PanEco, Russia) was added per well, and incubation proceeded with CO_2_ at 37 °C. Twenty-four hours after seeding the cells into wells, the tested compounds were added at a preset dilution, 150 μL to each well. Each experiment was repeated three times. Intact cells cultured in parallel with experimental cells were used as a control. The calculation of the concentration of the drug causing inhibition of cell growth by 50% (IC50) was performed with an Internet tool: MLA—“Quest Graph™ IC50 Calculator” (AAT Bioquest, Inc., https://www.aatbio.com/tools/ic50-calculator, accessed on 25 July 2019).

## 4. Conclusions

Thus, for the first time, we have synthesized a series of water-soluble sulfobetaine derivatives of *p-tert*-butylthiacalix[4]arene in *cone*, *partial cone*, and *1,3-alternate* conformation. It was shown by dynamic light scattering that none of the synthesized macrocycles **3**–**5** form stable self-associates in water. The association of macrocycles **3**–**5** with Ag^+^ was confirmed by DLS methods. The particle size and polydispersity of systems depend on the conformation of macrocycle and are determined by the spatial structure of the sulfobetaine fragments. It was shown by ^1^H and ^1^H-^1^H NOESY NMR spectroscopy that in the case of *cone* and *1,3-alternate*, intramolecular electrostatic attraction in the sulfobetaine fragment becomes possible, leading to the formation of a stable six-membered ring and coordination of Ag^+^ exclusively over the bridging sulfur atoms and the carbonyl oxygen atoms. In the case of a more conformationally mobile, asymmetric *partial cone* molecule, such charge compensation turns out to be impossible, and a third coordination center (the terminal sulfo group) appears. This leads to a difference in the morphology of the formed aggregates. In the case of **4**/Ag^+^ aggregates, extended nanostructures are formed. The cytotoxic effects are shown to be controlled by the shape of the associates. Among the tested compounds, only the **4**/Ag^+^ aggregates act selectively on the cervical carcinoma cell line (M-HeLa). In terms of cytotoxic activity, this complex is two times higher than the reference drug imatinib mesylate. The selective activity against tumor cells in combination with low toxicity toward normal cells allow the consideration of **4**/Ag^+^ aggregates as effective novel antitumor agents.

We show structural requirements implicated in the anticancer activity of the thiacalix[4]arene/Ag+ system, which might help to rationalize their development as antitumor agents. We hope that the results of our work will make it possible to develop fundamentally new approaches to the synthesis of nanostructures without a drug to solve the problem of multidrug resistance. This work may become the basis for the creation of a new class of anticancer systems: "nano anticancer drugs”. The directed design of the structures allows the control of the activity, selectivity, and biological compatibility of nanoparticles exhibiting anticancer properties.

## Figures and Tables

**Table 1:** In vitro cytotoxic effects (μM) of sulfobetaine derivatives **3**–**5** and their aggregates with Ag^+^.

Test Compounds	IC_50_ (µM)			
	M-HeLa	HuTu 80	MCF-7	Chang Liver
**3**	>5000	>5000	>5000	>5000
**3/**Ag^+^	>5000	>5000	>5000	>5000
**4**	>500	>500	>500	>500
**4/**Ag^+^	**38.9 ± 2.6**	>500	>500	**85.1 ± 6.4**
**5**	>500	>500	>500	>500
Imatinib mesylate	84.7 ± 6.3	288 ± 23	207 ± 17	102 ± 7.9

The experiments were repeated for three times. The results are expressed as the mean ± standard deviation.

## Data Availability

The data presented in this study are available in Supplementary Materials.

## Supplementary Materials

The following are available online, Figures S1, S5 and S9: ^1^H NMR spectra of the compounds **3**–**5**; Figures S2, S6 and S10: ^13^C NMR spectra of the compounds **3**–**5**; Figures S3, S7 and S11: Mass spectra of the compounds **3**–**5**; Figures S4, S8 and S12: IR spectra of the compounds **3**–**5**; Figures S13–S42: Size distributions of compounds **3**–**5** and mixture with Ag^+^; Figures S43–S45: UV spectra of compounds **3**–**5** with Ag^+^; Figures S4 and S47: ^1^H NMR spectrum of the compounds **3** and **5** and their mixtures with Ag^+^; Figures S48–S53: 2D ^1^H–^1^H NOESY NMR spectra of the compounds **3**–**5** and their mixtures with Ag^+^; Tables S1–S4: Size of aggregates formed during association of compound **3**–**5** and their mixtures with Ag^+^.

## Sample Availability

Samples of all obtained compounds are available from the authors.
