# Supplementary material for: Structure–Activity Relationship of the Thiacalix[4]arenes Family with Sulfobetaine Fragments: Self-Assembly and Cytotoxic Effect against Cancer Cell Lines
Source: Molecules. 2022 Feb 17;27(4):1364. doi: 10.3390/molecules27041364 (PMC8879733; doi:10.3390/molecules27041364)
Supplement: Supplementary file 1 [file molecules-27-01364-s001.zip › molecules-1592699-Supplementary materials.pdf]

Structure–Activity Relationship of the Thiacalix[4]arenes Family with Sulfobetaine Fragments: Self-Assembly and Cytotoxic Effect against Cancer Cell Lines

Luidmila Yakimova [1,*], Aisylu Kunafina [1], Aigul Nugmanova [1], Pavel Padnya [1], Alexandra Voloshina [2], Konstantin Petrov [2] and Ivan Stoikov [1,*]

[1] A.M. Butlerov' Chemistry Institute, Kazan Federal University, 18 Kremlevskaya Street, 420008 Kazan, Russia; ais.kunaf@yandex.ru (A.K.); aygul9pul9@mail.ru (A.N.); padnya.ksu@gmail.com (P.P.)
[2] Arbuzov Institute of Organic and Physical Chemistry, FRC Kazan Scientific Center of RAS, 8 Arbuzov Street, 420088 Kazan, Russia; microbi@iopc.ru (A.V.); kpetrov2005@mail.ru (K.P.)
* Correspondence: mila.yakimova@mail.ru (L.Y.); ivan.stoikov@mail.ru (I.S.); Tel.: +7-843-233-7241 (I.S.)

Electronic Supplementary Information (33 pages)

1. NMR, ESI, IR spectra of compounds **3-5**.
2. Dynamic light scattering
3. UV spectra
4. NMR spectra of associates
5. Tables

1. NMR, ESI, IR spectra of compounds 3-5

General procedure for the synthesis of compound **3-5**

The mixture of 5,11,17,23-tetra-*tert*-butyl-25,26,27,28-tetrakis[(N-(3',3'-dimethylaminopropyl)-carbamoylmethoxy]-2,8,14,20-tetrathiacalix[4]arene **(2-*cone*, 2-*paco*, 2-*alt*)** (0.20 g, 0.16 mmol) and 1,3-propanesultone (0.64 mmol) in 15 ml of solvent (CH_3CN) was refluxed for 72 hours. After cooling, solvent was evaporated under reduce pressure.

5,11,17,23-Tetra-*tert*-butyl-25,26,27,28-tetrakis[(N-(3',3'-dimethyl-3'-{3"-sulfonatopropyl})ammoniumpropyl)-carbamoylmethoxy]-2,8,14,20-tetrathiacalix[4]arene (*cone*) **(3)**. Yield 0.19 g (79 %), Mp: 258°C. ^{1}H NMR (DMSO-d_6, δ, ppm, *J*/Hz): 1.07 (s, 36H, $(CH_3)_3C$), 2.01 (m, 8H, $-NCH_2\underline{CH_2}CH_2NH$), 1.95 (m, 8H, $-N\underline{CH_2}CH_2CH_2SO_3^-$), 2.01 (m, 8H, -$NCH_2\underline{CH_2}CH_2NH$), 2.52 (m, 8H, $-NCH_2CH_2\underline{CH_2}SO_3^-$), 3.04 (s, 24H, $(CH_3)_2N^+$), 3.26 (m, 8H, $N\underline{CH_2}CH_2CH_2NH$), 3.36 (m, 8H, $-N\underline{CH_2}CH_2CH_2SO_3^-$), 3.44 (m, 8H, $NCH_2CH_2\underline{CH_2}NH$), 4.84 (s,

8H, OCH_2CO), 7.40 (s, 8H, ArH), 8.52 (br. s, 4H, CONH). ^{13}C NMR (DMSO-d_6, δ, ppm): 68.24, 157.82, 146.70, 134.51, 128.15, 74.11, 61.98, 60.73, 50.30, 47.74, 35.50, 33.97, 30.76, 22.44, 18.89. El. Anal. Calcd for $C_{80}H_{128}N_8O_{20}S_8$ (%): C 54.03 %, H 7.25 %, N 6.30 %, S 14.42 %. Found (%): C 54.50 %, H 7.18 %, N 6.59 %, S 14.45 %. FTIR ATR (ν,cm^{-1}): 1663 (C=O), 2958 (N+), 3322 (NH). MS (ESI): calculated 1776.8 $[M+2Na]^{2+}$, found: 1776.3 $[M+2Na]^{2+}$.

5,11,17,23-Tetra-*tert*-butyl-25,26,27,28-tetrakis[(N-(3',3'-dimethyl-3'-{3"-sulfonatopropyl})ammoniumpropyl)-carbamoylmethoxy]-2,8,14,20-tetrathiacalix[4]arene (*partial cone*) **(4)**. Yield 0.26 g (95 %). ^1H NMR (DMSO-d_6, δ, ppm, *J*/Hz): 1.01 (s, 18H, $(CH_3)_3C$), 1.28 (s, 9H, $(CH_3)_3C$), 1.29 (s, 9H, $(CH_3)_3C$), 1.80-2.04 (m, 16H, -$N^+CH_2\underline{CH_2}CH_2SO_3^-$, -$NCH_2\underline{CH_2}CH_2NH$), 2.45 (m, 8H, -$N^+CH_2CH_2\underline{CH_2}SO_3^-$), 2.97-3.03 (m, 24H, $(CH_3)_2N^+$), 3.14-3.30 (m, 16H, $N^+\underline{CH_2}CH_2CH_2NH$, -$N^+\underline{CH_2}CH_2CH_2SO_3^-$), 3.4 (m, 8H, $N^+CH_2CH_2\underline{CH_2}NH$), 4.36 (d, 2H, OCH_2CO, $^2J_{HH}$=13.6 Hz), 4.46 (s, 2H, OCH_2CO), 4.54 (s, 2H, OCH_2CO), 4.82 (d, 2H, OCH_2CO, $^2J_{HH}$=13.6 Hz), 7.02 (m, 2H, ArH), 7.64 (m, 2H, ArH), 7.67 (s, 2H, ArH),7.76 (s, 2H, ArH), 8.32 (m, 2H, CONH), 8.45 (m, 2H, CONH). ^{13}C NMR (DMSO-d_6, δ, ppm): 169.21, 168.40, 167.51, 160.02, 157.70, 147.04, 146.08, 135.92, 135.31, 134.44, 128.48, 127.95, 127.49, 126.92, 73.17, 63.07, 62.67, 61.46, 61.35, 50.49, 48.09, 36.09, 34.45, 34.37, 34.28, 31.48, 31.19, 22.76, 22.68, 19.32. FTIR ATR (ν,cm^{-1}): 1663 (C=O), 2954 (N^+), 3429 (NH). El. Anal. Calcd for $C_{80}H_{128}N_8O_{20}S_8$ (%): C 54.03 %, H 7.25 %, N 6.30 %, S 14.42 %. Found (%): C 54.47 %, H 7.17 %, N 6.57 %, S 14.41 %. MS (ESI): calculated 911.339 $[M+2Na]^{2+}$, 615.222 $[M+3Na]^{3+}$, 607.895 $[M+2Na+H]^{3+}$, found: 911.337 $[M+2Na]^{2+}$, 615.221 $[M+3Na]^{3+}$, 607.893 $[M+2Na+H]^{3+}$.

5,11,17,23-Tetra-*tert*-butyl-25,26,27,28-tetrakis[(N-(3',3'-dimethyl-3'-{3"-sulfonatopropyl})ammoniumpropyl)-carbamoylmethoxy]-2,8,14,20-tetrathiacalix[4]arene (*1,3-alternate*) **(5)**. Yield 0.25 g (91 %). ^1H NMR (DMSO-d_6, δ, ppm, *J*/Hz): 1.20 (s, 36H, $(CH_3)_3C$), 1.89 (m, 8H, -$NCH_2\underline{CH_2}CH_2NH$), 1.99 (m, 8H, -$N^+CH_2\underline{CH_2}CH_2SO_3^-$), 2.06 (m, 8H, -$N^+CH_2CH_2\underline{CH_2}SO_3^-$), 3.01 (s, 24H, $(CH_3)_2N^+$), 3.18 (m, 8H, -$N^+\underline{CH_2}CH_2CH_2SO_3^-$), 3.30 (m, 8H, $NCH_2CH_2\underline{CH_2}NH$), 3.43 (m, 8H, $N\underline{CH_2}CH_2CH_2NH$), 4.01 (s, 8H, OCH_2CO), 7.60 (s, 8H, ArH), 8.06 (m, 4H, CONH). ^{13}C NMR (DMSO-d_6, δ, ppm): 167.79, 157.64, 146.54, 133.50, 127.98, 71.54, 62.84, 61.39, 50.52, 48.09, 39.90, 36.19, 34.37, 31.27, 22.84, 19.32. FTIR ATR (ν,cm^{-1}): 1657 (C=O), 2957 (N^+) 3300 (NH). El. Anal. Calcd for $C_{80}H_{128}N_8O_{20}S_8$ (%): C 54.03 %, H 7.25 %, N 6.30 %, S 14.42 %. Found (%): C 54.36 %, H 7.28 %, N 6.47 %, S 14.40 %. MS (ESI): calculated 1777.7 $[M+H]^+$, 889.4 $[M+2H]^{2+}$, found: 1777.6 $[M+H]^+$, 889.8 $[M+2H]^{2+}$.

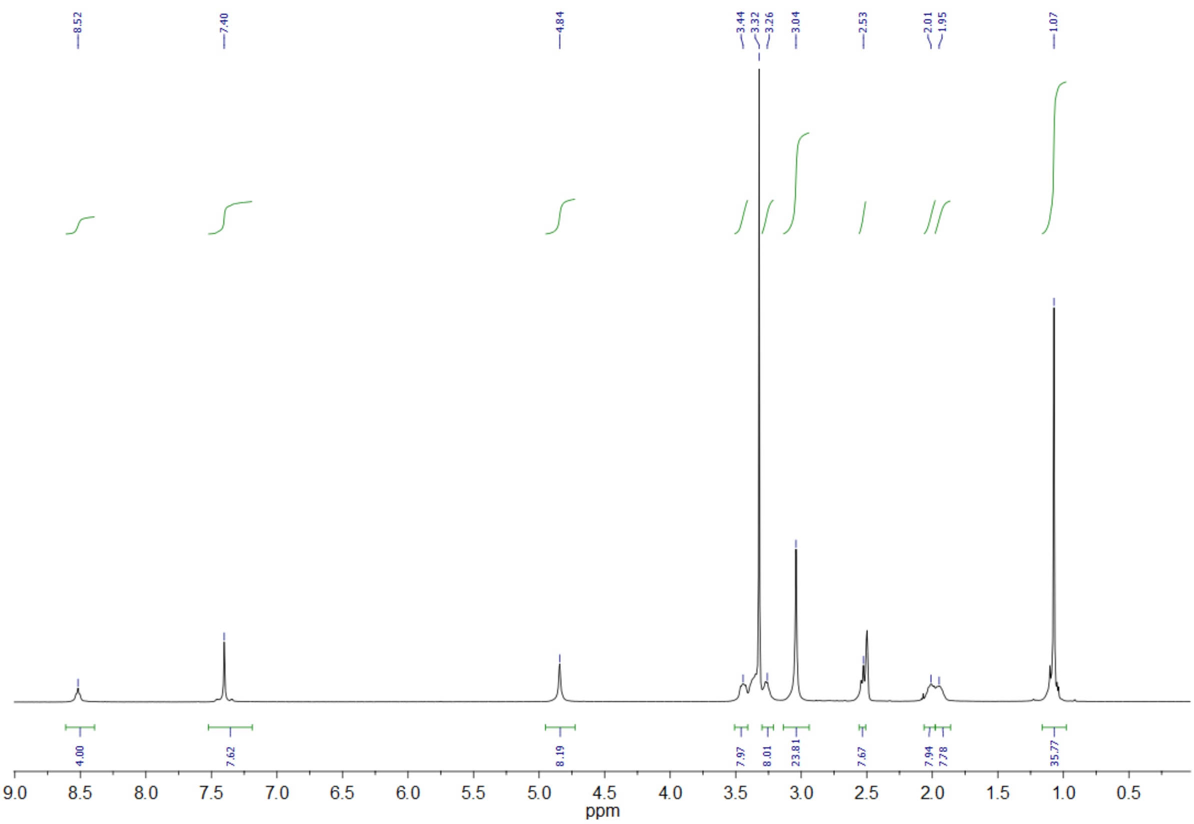

Figure S1. ^{1}H NMR spectrum of 5,11,17,23-tetra-*tert*-butyl-25,26,27,28-tetrakis[(N-(3′,3′-dimethyl- 3′-{3″-sulfonatopropyl})ammoniumpropyl)-carbamoylmethoxy]-2,8,14,20-tetrathiacalix[4]arene(*cone*) **(3)**, DMSO-d_6, 298 K, 400 MHz.

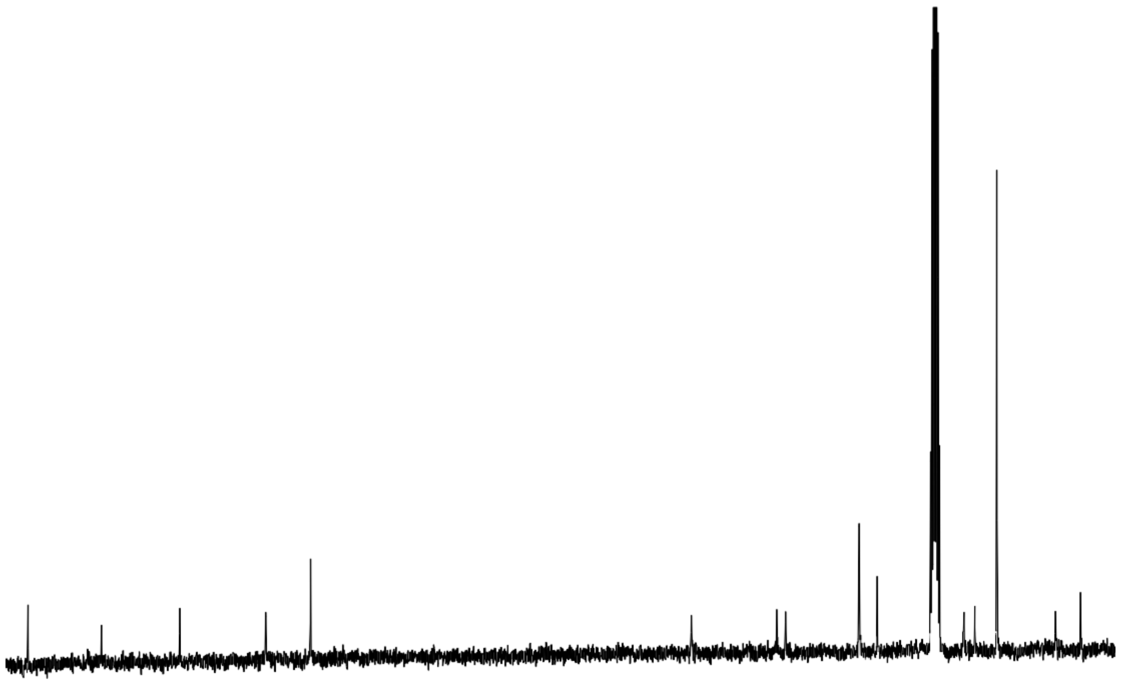

Figure S2. ^{13}C NMR spectrum of 5,11,17,23-tetra-*tert*-butyl-25,26,27,28-tetrakis[(N-(3’,3’-dimethyl-3’-{3”-sulfonatopropyl})ammoniumpropyl)-carbamoylmethoxy]-2,8,14,20-tetrathiacalix[4]arene (*cone*) **(3)**, DMSO-d_6, 298 K, 400 MHz.

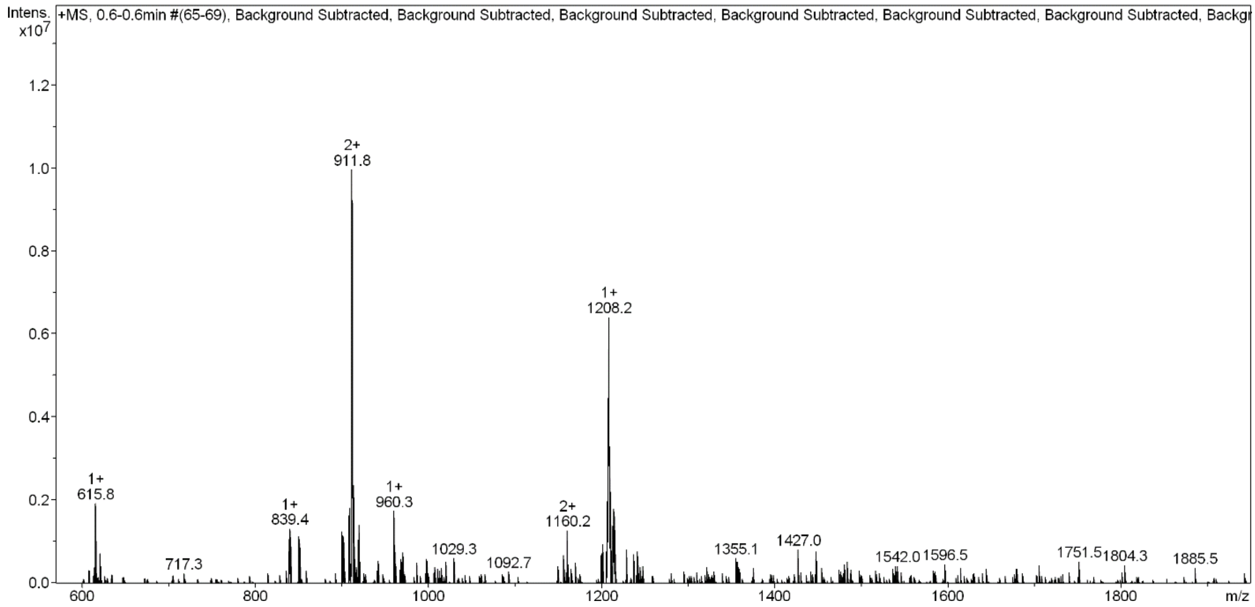

Figure S3. Mass spectrum (ESI) of 5,11,17,23-tetra-*tert*-butyl-25,26,27,28-tetrakis[(N-(3′,3′-dimethyl-3’-{3”-sulfonatopropyl})ammoniumpropyl)-carbamoylmethoxy]-2,8,14,20-tetrathiacalix[4]arene (*cone*) **(3)**.

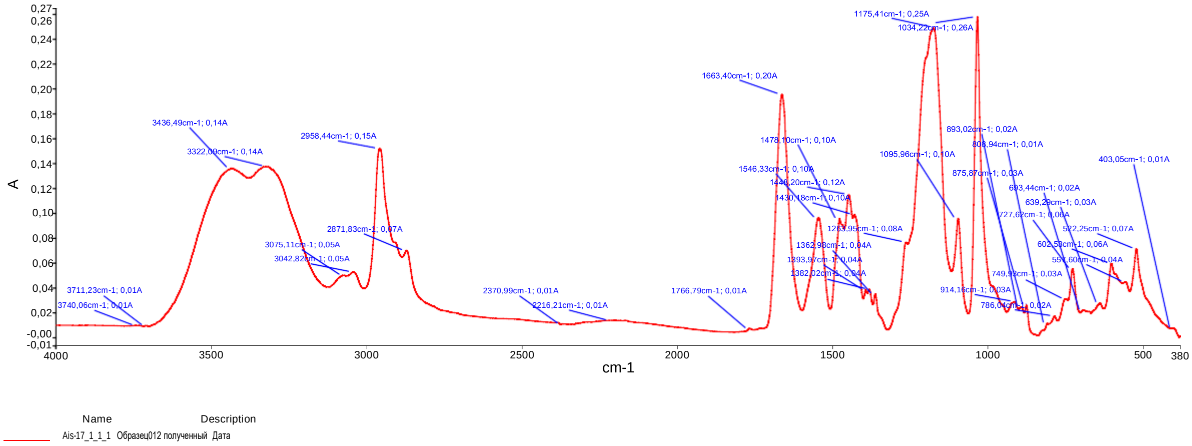

Figure S4. IR spectrum of 5,11,17,23-tetra-*tert*-butyl-25,26,27,28-tetrakis[(N-(3′,3′-dimethyl-3′-{3″-sulfonatopropyl})ammoniumpropyl)-carbamoylmethoxy]-2,8,14,20-tetrathiacalix[4]arene (*cone*) **(3)**.

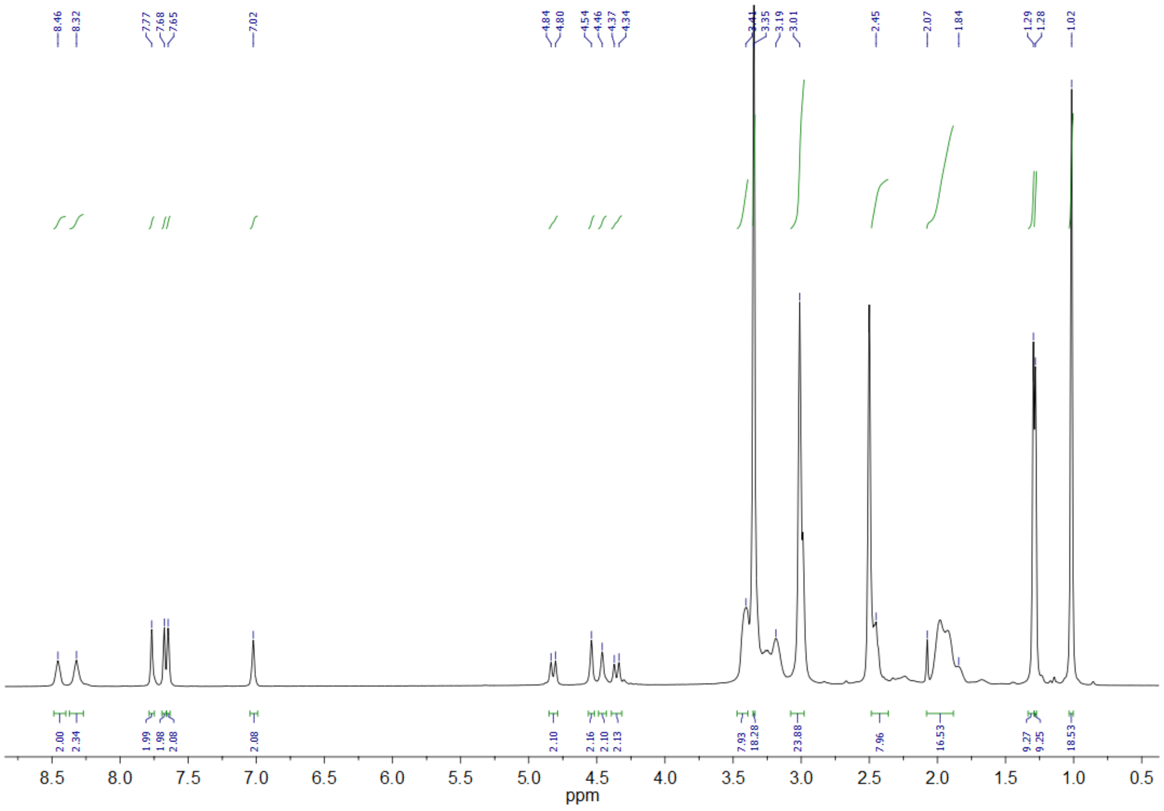

Figure S5. ^{1}H NMR spectrum of 5,11,17,23-tetra-*tert*-butyl-25,26,27,28-tetrakis[(N-(3′,3′-dimethyl- 3′-{3″-sulfonatopropyl})ammoniumpropyl)-carbamoylmethoxy]-2,8,14,20-tetrathiacalix[4]arene(*partial cone*) **(4)**, DMSO-d_6, 298 K, 400 MHz.

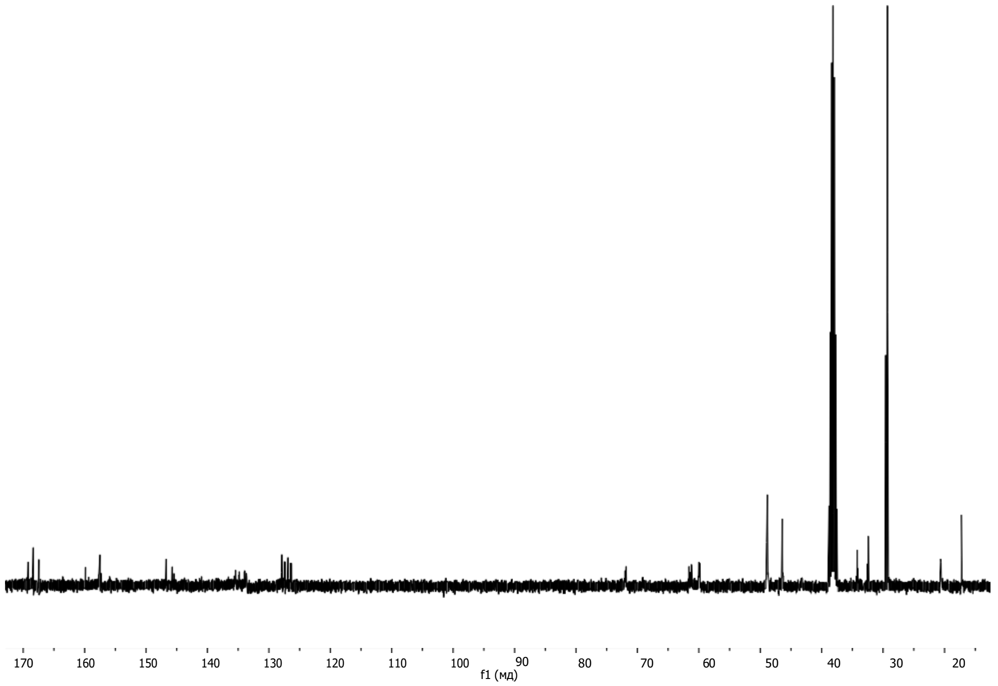

Figure S6. ^{13}C NMR spectrum of 5,11,17,23-tetra-*tert*-butyl-25,26,27,28-tetrakis[(N-(3′,3′-dimethyl-3′-{3″-sulfonatopropyl})ammoniumpropyl)-carbamoylmethoxy]-2,8,14,20-tetrathiacalix[4]arene (*partial cone*) **(4)**, DMSO-d_6, 298 K, 400 MHz.

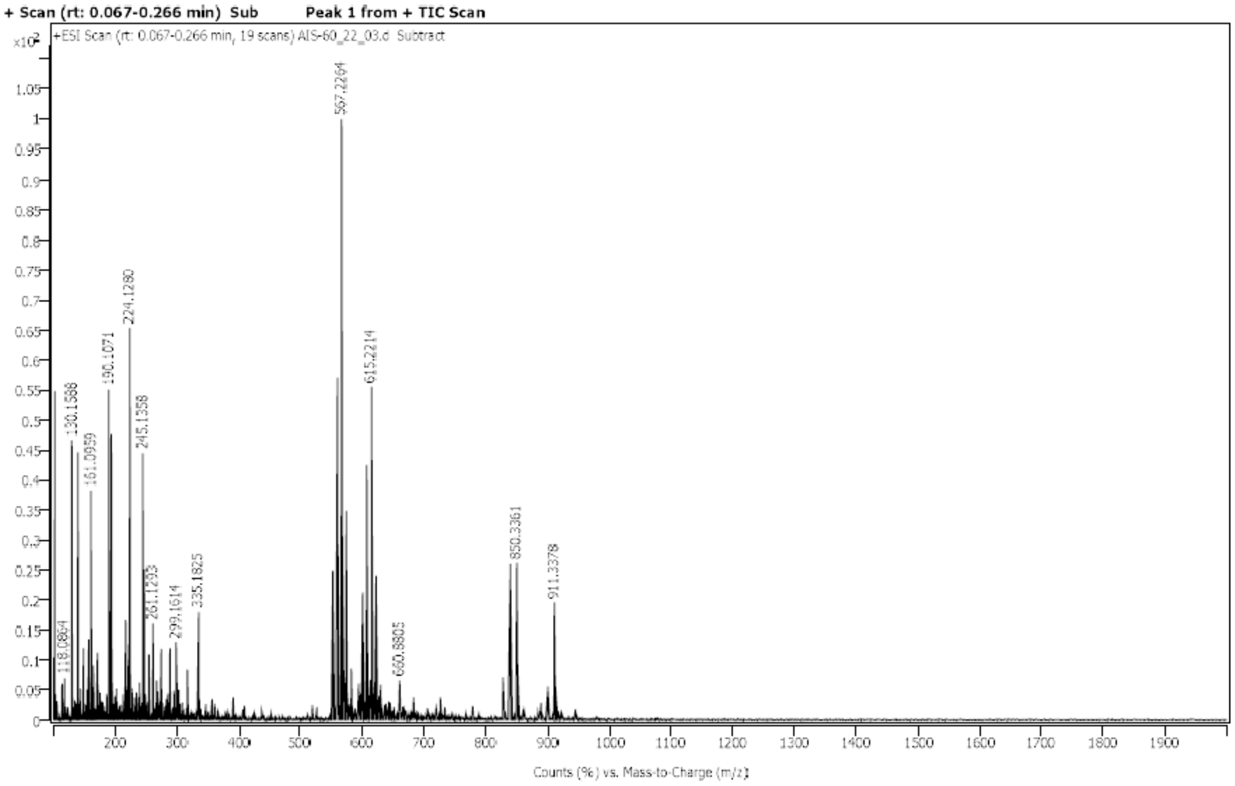

Figure S7. Mass spectrum (ESI) of 5,11,17,23-tetra-*tert*-butyl-25,26,27,28-tetrakis[(N-(3′,3′-dimethyl-3′-{3″-sulfonatopropyl})ammoniumpropyl)-carbamoylmethoxy]-2,8,14,20-tetrathiacalix[4]arene (*partial cone*) **(4)**.

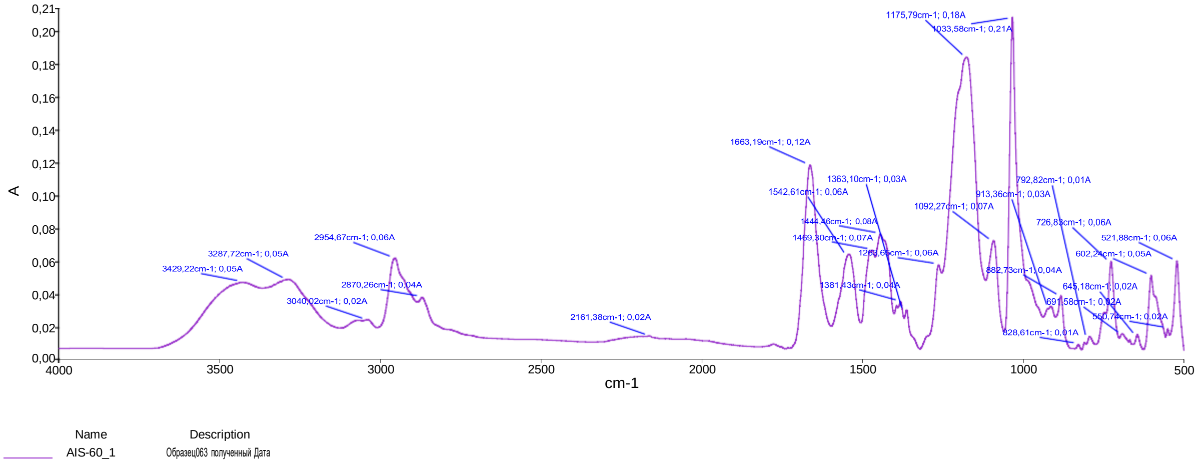

Figure S8. IR spectrum of 5,11,17,23-tetra-*tert*-butyl-25,26,27,28-tetrakis[(N-(3′,3′-dimethyl-3′-{3″-sulfonatopropyl})ammoniumpropyl)-carbamoylmethoxy]-2,8,14,20-tetrathiacalix[4]arene (*partial cone*) **(4)**.

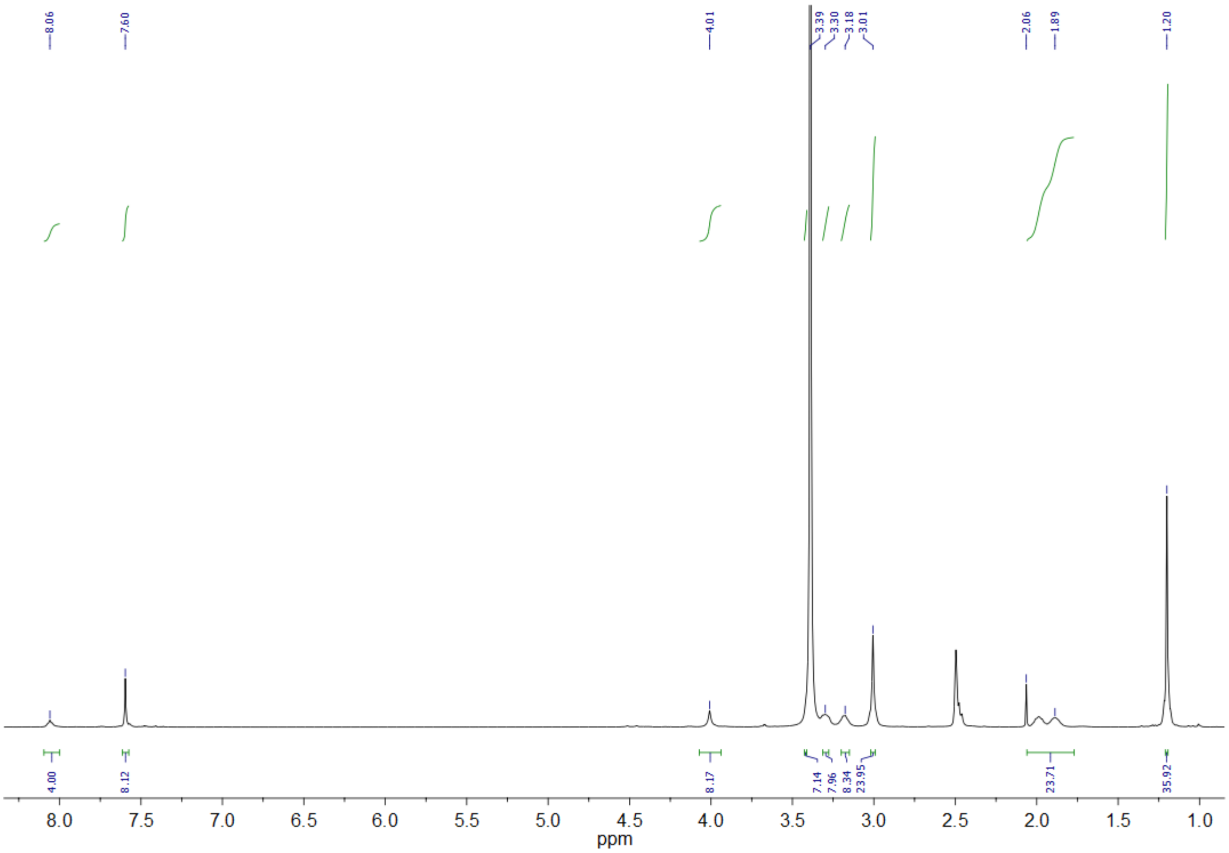

Figure S9. ^{1}H NMR spectrum of 5,11,17,23-tetra-*tert*-butyl-25,26,27,28-tetrakis[(N-(3′,3′-dimethyl- 3′-{3″-sulfonatopropyl})ammoniumpropyl)-carbamoylmethoxy]-2,8,14,20-tetrathiacalix[4]arene(*1,3-alternate*) **(5)**, DMSO-d_6, 298 K, 400 MHz.

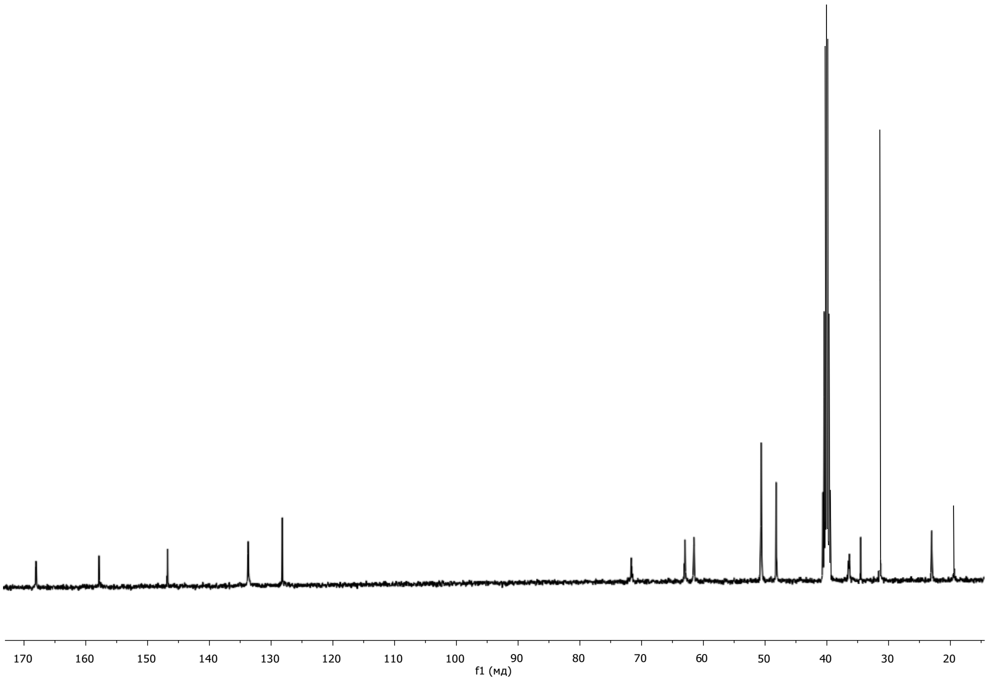

Figure S10. ^{13}C NMR spectrum of 5,11,17,23-tetra-*tert*-butyl-25,26,27,28-tetrakis[(N-(3′,3′-dimethyl-3′-{3″-sulfonatopropyl})ammoniumpropyl)-carbamoylmethoxy]-2,8,14,20-tetrathiacalix[4]arene (*1,3-alternate*) **(5)**, DMSO-d_6, 298 K, 400 MHz.

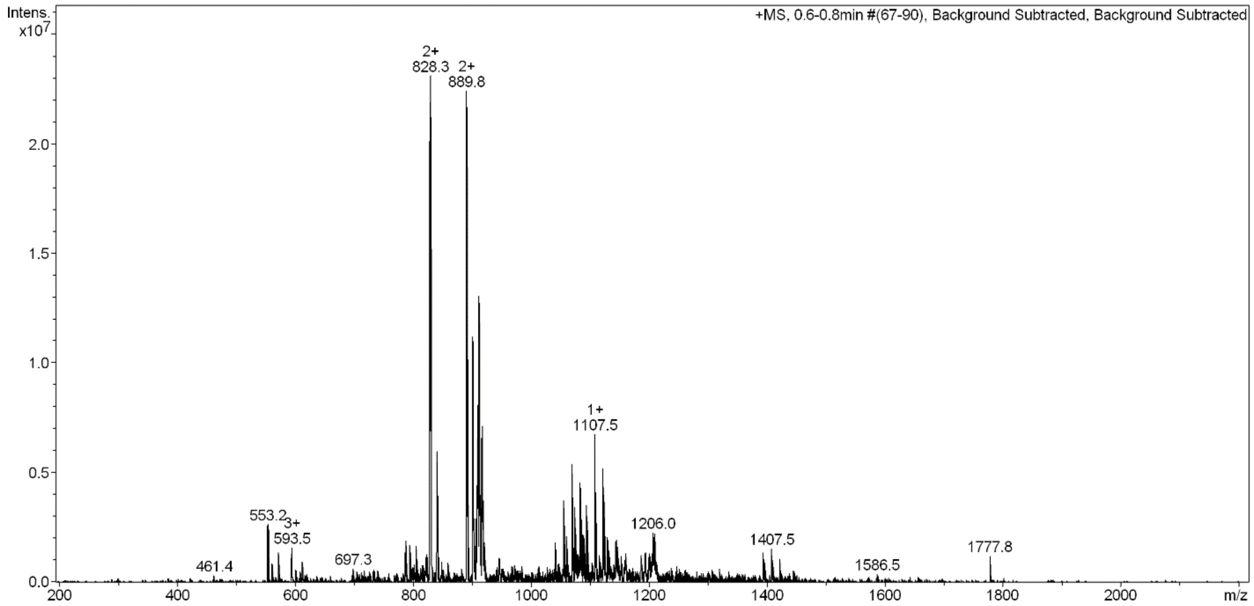

Figure S11. Mass spectrum (ESI) of 5,11,17,23-tetra-*tert*-butyl-25,26,27,28-tetrakis[(N-(3′,3′-dimethyl-3′-{3″-sulfonatopropyl})ammoniumpropyl)-carbamoylmethoxy]-2,8,14,20-tetrathiacalix[4]arene (*1,3-alternate*) **(5)**.

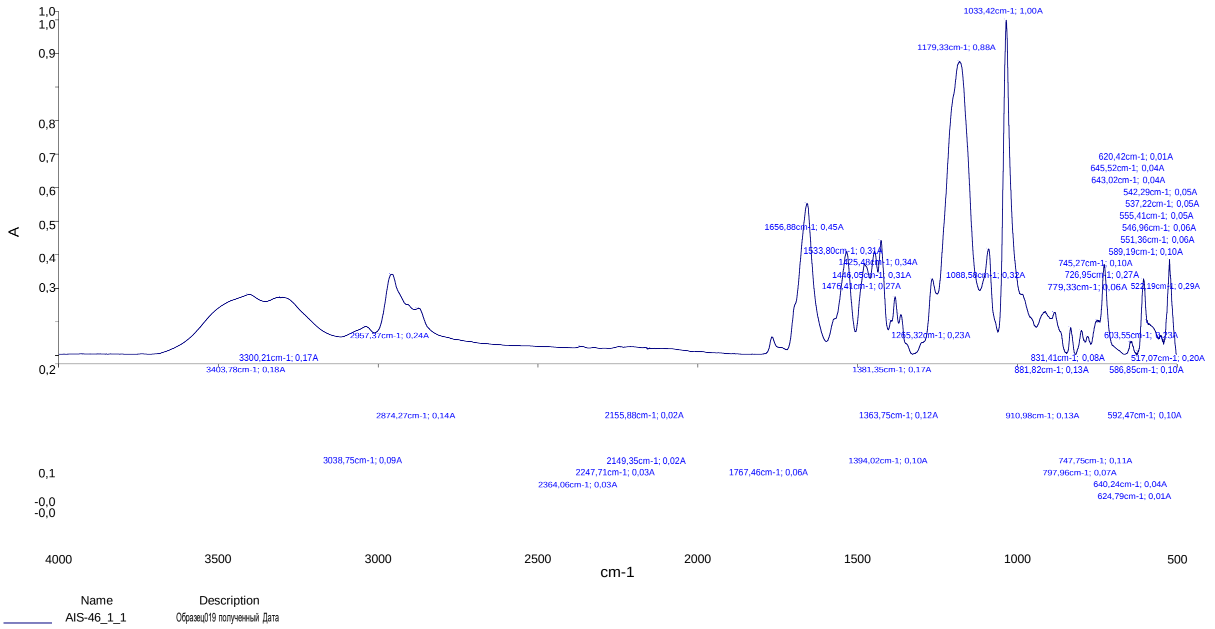

Figure S12. IR spectrum of 5,11,17,23-tetra-*tert*-butyl-25,26,27,28-tetrakis[(N-(3′,3′-dimethyl-3′-{3″-sulfonatopropyl})ammoniumpropyl)-carbamoylmethoxy]-2,8,14,20-tetrathiacalix[4]arene (*1,3-alternate*) **(5)**.

2. Dynamic light scattering

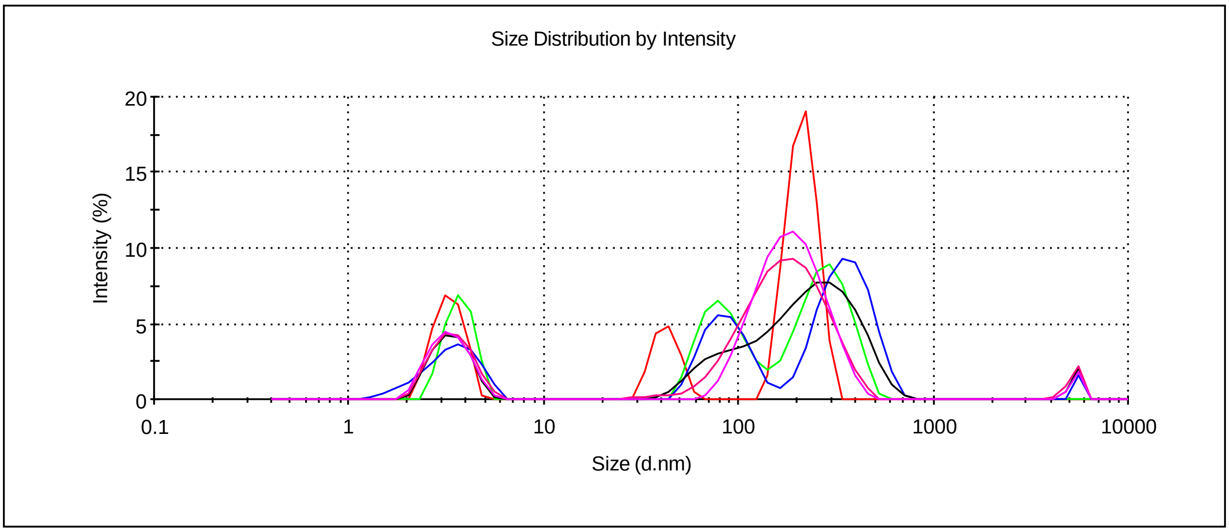

Figure. S13. Size distribution of 5,11,17,23-tetra-*tert*-butyl-25,26,27,28-tetrakis[(N-(3′,3′-dimethyl- 3′-{3″-sulfonatopropyl})ammoniumpropyl)-carbamoylmethoxy]-2,8,14,20-tetrathiacalix[4]arene(*cone*) **(3)** in water (3 × 10^{-4} M).

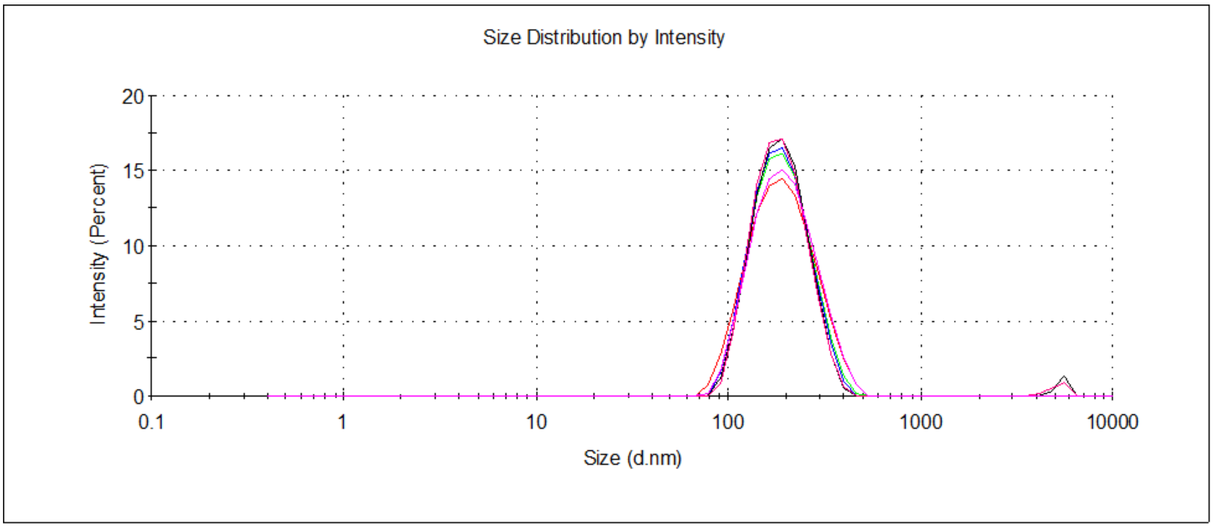

Figure S14. Size distribution of 5,11,17,23-tetra-*tert*-butyl-25,26,27,28-tetrakis[(N-(3′,3′-dimethyl- 3′-{3″-sulfonatopropyl})ammoniumpropyl)-carbamoylmethoxy]-2,8,14,20-tetrathiacalix[4]arene(*cone*) **(3)** in the presence of Ag^{I} in water (3 × 10^{-4} M), (1:1).

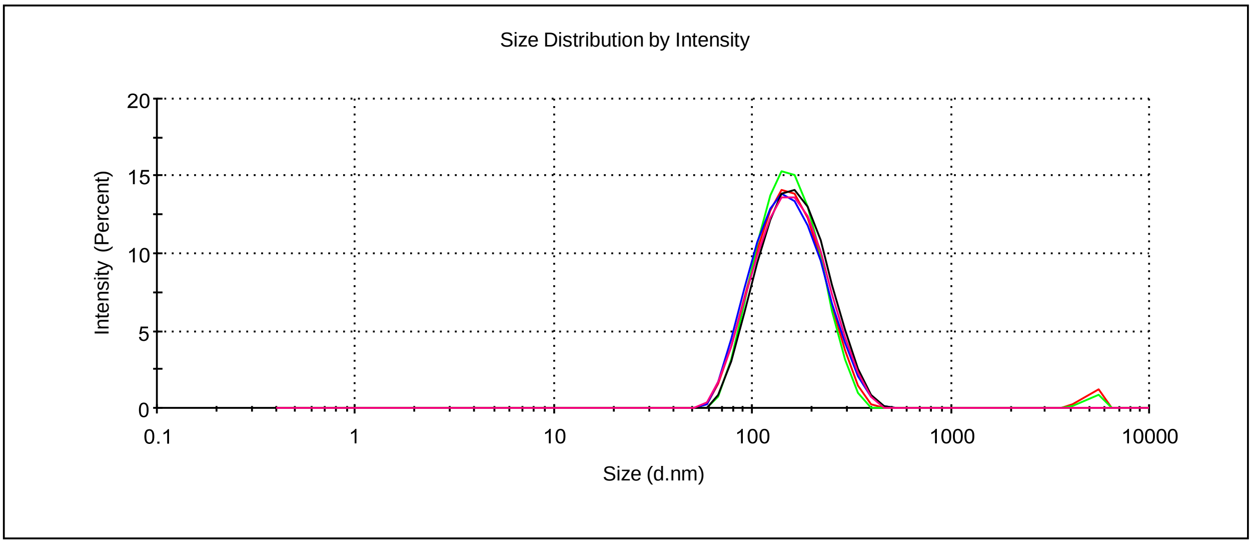

Figure S15. Size distribution of 5,11,17,23-tetra-*tert*-butyl-25,26,27,28-tetrakis[(N-(3′,3′-dimethyl- 3′-{3″-sulfonatopropyl})ammoniumpropyl)-carbamoylmethoxy]-2,8,14,20-tetrathiacalix[4]arene(*cone*) **(3)** in the presence of Ag^{I} in water (1×10^{-4} M), (1:1).

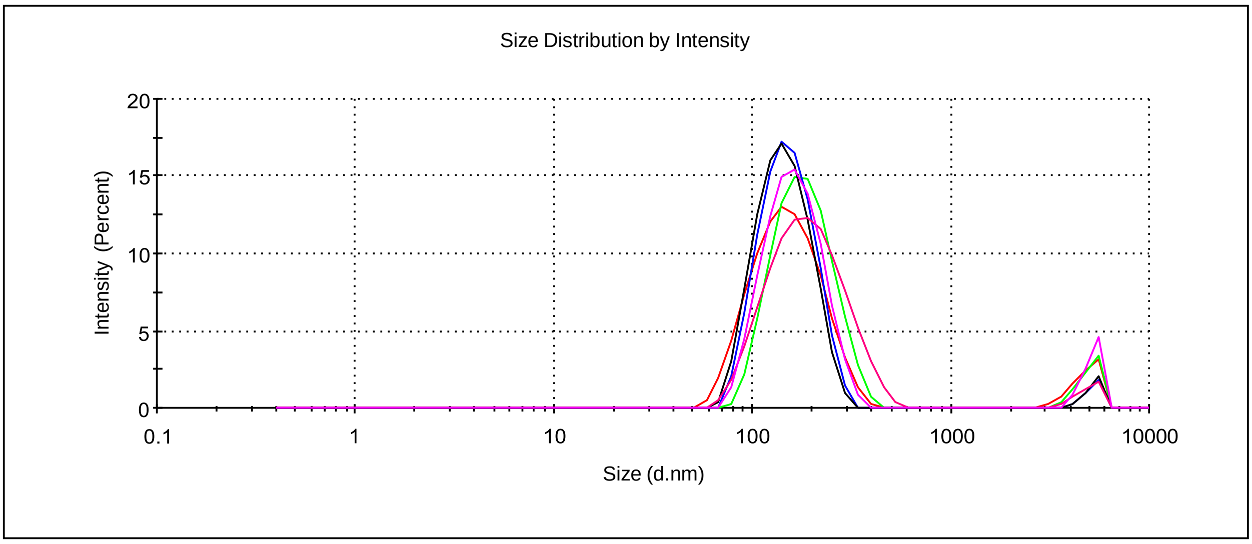

Figure S16. Size distribution of 5,11,17,23-tetra-*tert*-butyl-25,26,27,28-tetrakis[(N-(3′,3′-dimethyl- 3′-{3″-sulfonatopropyl})ammoniumpropyl)-carbamoylmethoxy]-2,8,14,20-tetrathiacalix[4]arene(*cone*) **(3)** in the presence of Ag^{I} in water ($1 \cdot \times 10^{-5}$ M), (1:1).

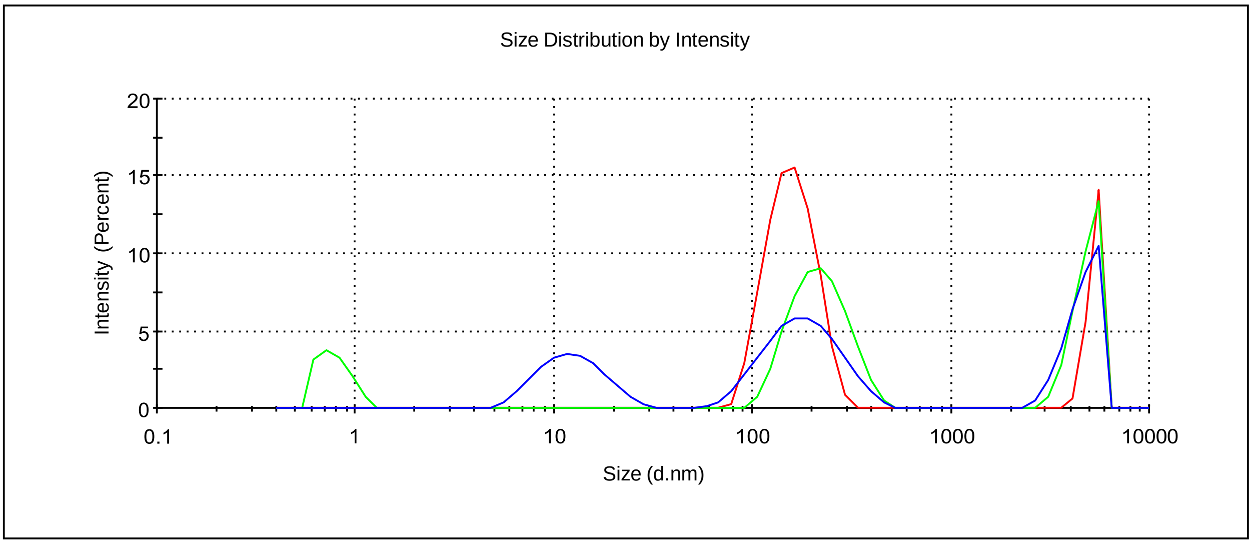

Figure S17. Size distribution of 5,11,17,23-tetra-*tert*-butyl-25,26,27,28-tetrakis[(N-(3′,3′-dimethyl- 3′-{3″-sulfonatopropyl})ammoniumpropyl)-carbamoylmethoxy]-2,8,14,20-tetrathiacalix[4]arene(*cone*) **(3)** in the presence of Ag^{I} in water ($1\cdot\times 10^{-6}$ M), (1:1).

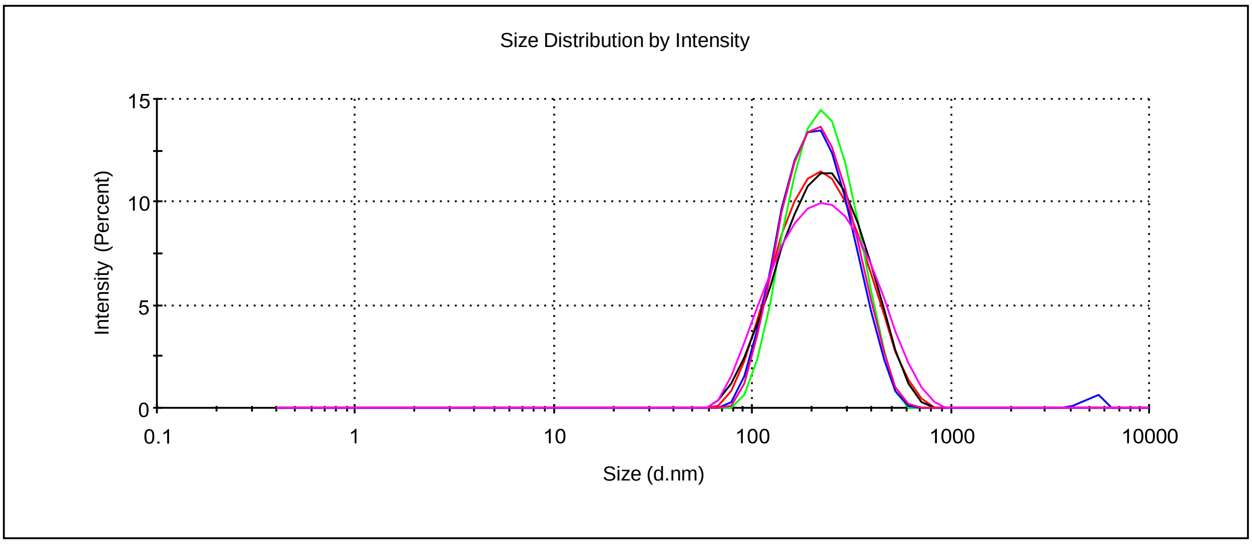

Figure S18. Size distribution of 5,11,17,23-tetra-*tert*-butyl-25,26,27,28-tetrakis[(N-(3′,3′-dimethyl- 3′-{3″-sulfonatopropyl}))ammoniumpropyl)-carbamoylmethoxy]-2,8,14,20-tetrathiacalix[4]arene(*cone*) **(3)** in the presence of Ag^{I} in water ($1\cdot\times 10^{-4}$ M), (1:4).

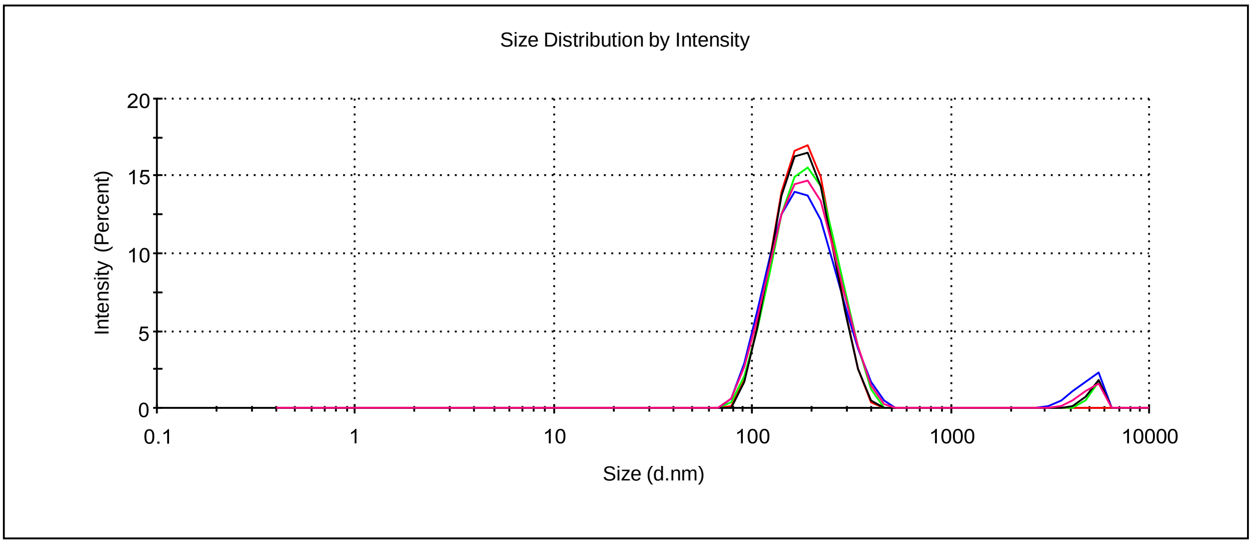

Figure S19. Size distribution of 5,11,17,23-tetra-*tert*-butyl-25,26,27,28-tetrakis[(N-(3′,3′-dimethyl- 3′-{3″-sulfonatopropyl}))ammoniumpropyl)-carbamoylmethoxy]-2,8,14,20-tetrathiacalix[4]arene(*cone*) **(3)** in the presence of Ag^{I} in water ($1 \cdot \times 10^{-5}$ M), (1:4).

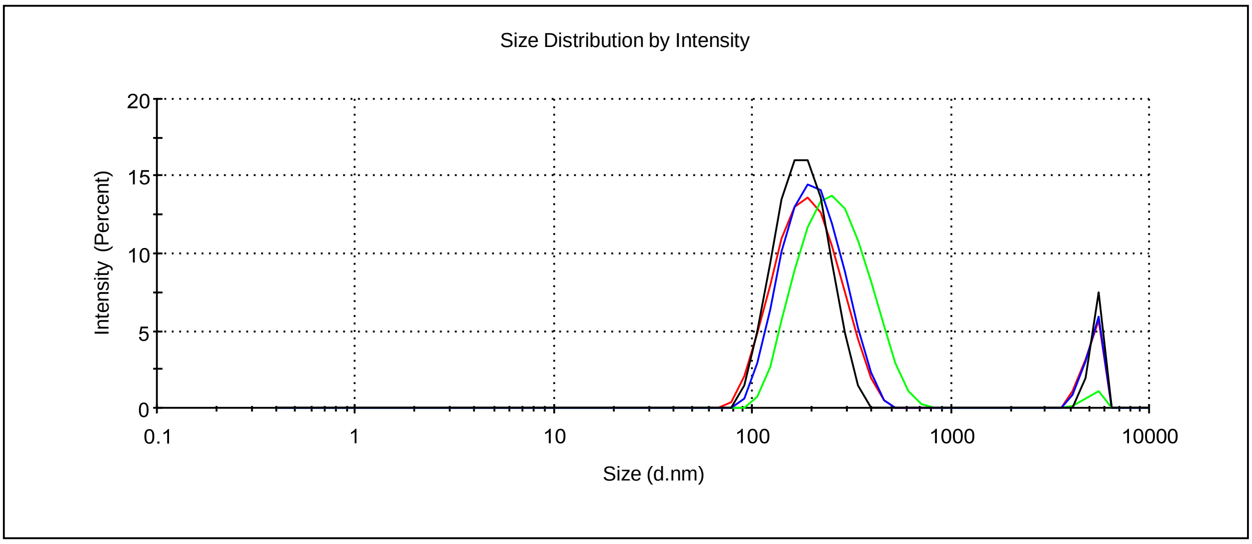

Figure S20. Size distribution of 5,11,17,23-tetra-*tert*-butyl-25,26,27,28-tetrakis[(N-(3′,3′-dimethyl- 3′-{3″-sulfonatopropyl})ammoniumpropyl)-carbamoylmethoxy]-2,8,14,20-tetrathiacalix[4]arene(*cone*) **(3)** in the presence of Ag^{I} in water ($1 \cdot \times 10^{-6}$ M), (1:4).

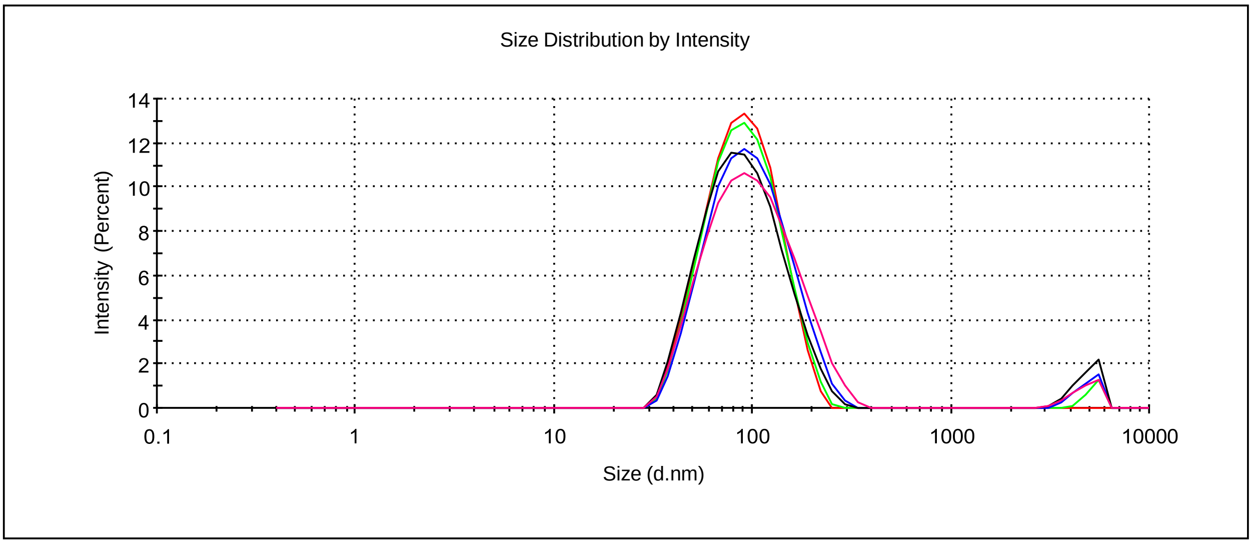

Figure S21. Size distribution of 5,11,17,23-tetra-*tert*-butyl-25,26,27,28-tetrakis[(N-(3′,3′-dimethyl- 3′-{3″-sulfonatopropyl})ammoniumpropyl)-carbamoylmethoxy]-2,8,14,20-tetrathiacalix[4]arene(*cone*) **(3)** in the presence of Ag^{I} in water ($1 \cdot \times 10^{-4}$ M), (1:10).

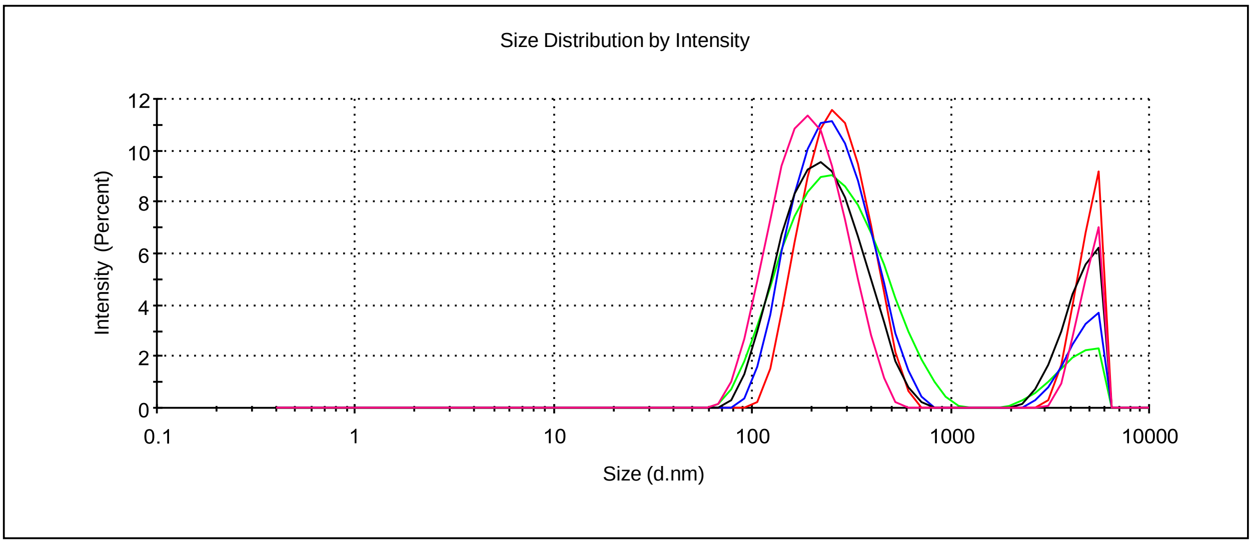

Figure S22. Size distribution of 5,11,17,23-tetra-*tert*-butyl-25,26,27,28-tetrakis[(N-(3′,3′-dimethyl- 3′-{3″-sulfonatopropyl})ammoniumpropyl)-carbamoylmethoxy]-2,8,14,20-tetrathiacalix[4]arene(*cone*) **(3)** in the presence of Ag^{I} in water ($1 \cdot \times 10^{-5}$ M), (1:10).

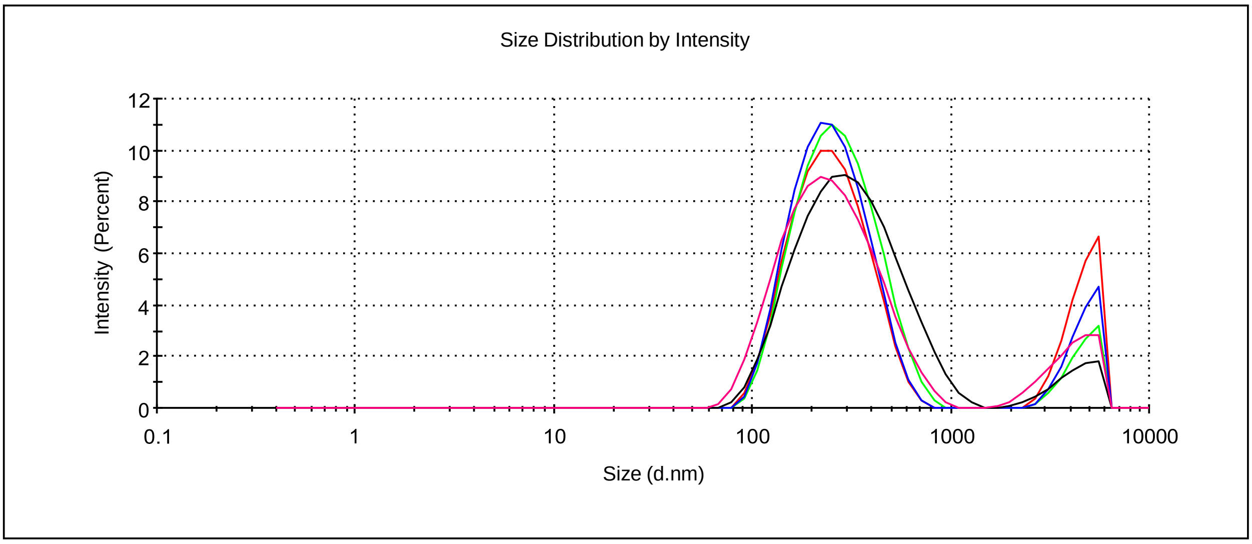

Figure S23. Size distribution of 5,11,17,23-tetra-*tert*-butyl-25,26,27,28-tetrakis[(N-(3′,3′-dimethyl-3′-{3″-sulfonatopropyl})ammoniumpropyl)-carbamoylmethoxy]-2,8,14,20-tetrathiacalix[4]arene(*cone*) **(3)** in the presence of Ag^{I} in water ($1\cdot\times10^{-6}$ M), (1:10).

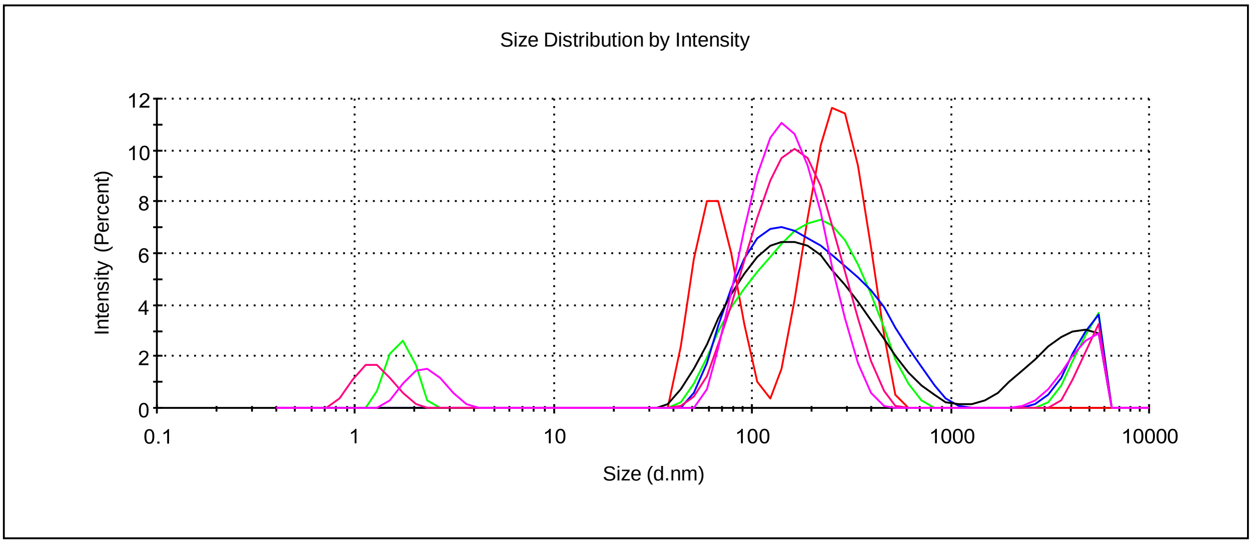

Figure S24. Size distribution of 5,11,17,23-tetra-*tert*-butyl-25,26,27,28-tetrakis[(N-(3′,3′-dimethyl-3′-{3″-sulfonatopropyl})ammoniumpropyl)-carbamoylmethoxy]-2,8,14,20-tetrathiacalix[4]arene(*partial cone*) **(4)** in water ($1\cdot\times10^{-4}$ M).

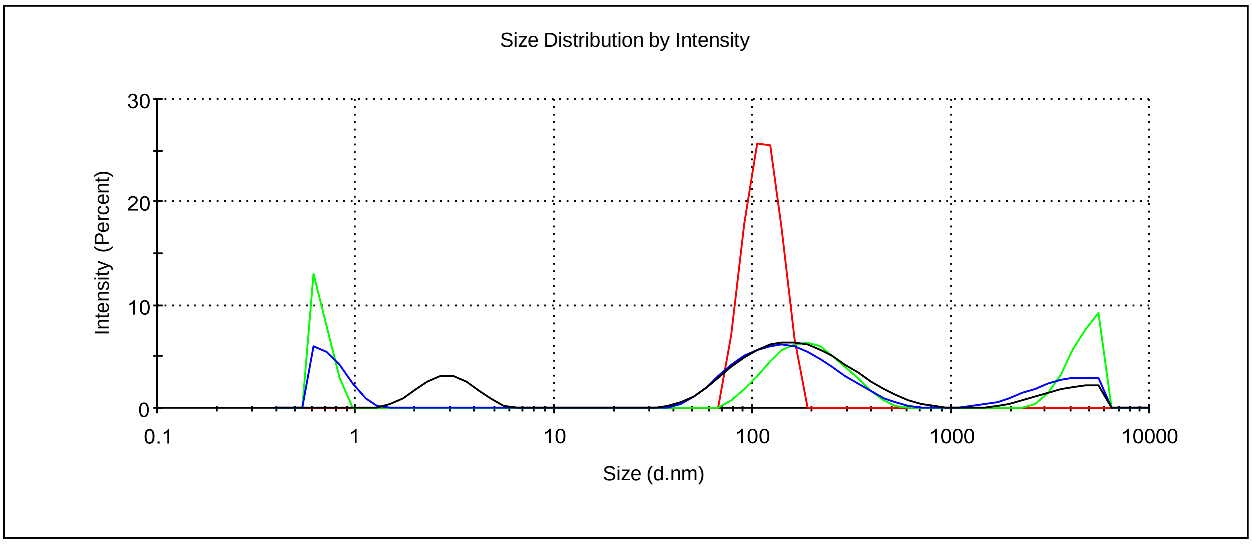

Figure S25. Size distribution of 5,11,17,23-tetra-*tert*-butyl-25,26,27,28-tetrakis[(N-(3′,3′-dimethyl- 3′-{3″-sulfonatopropyl})ammoniumpropyl)-carbamoylmethoxy]-2,8,14,20-tetrathiacalix[4]arene(*partial cone*) **(4)** in water ($1 \cdot \times 10^{-5}$ M).

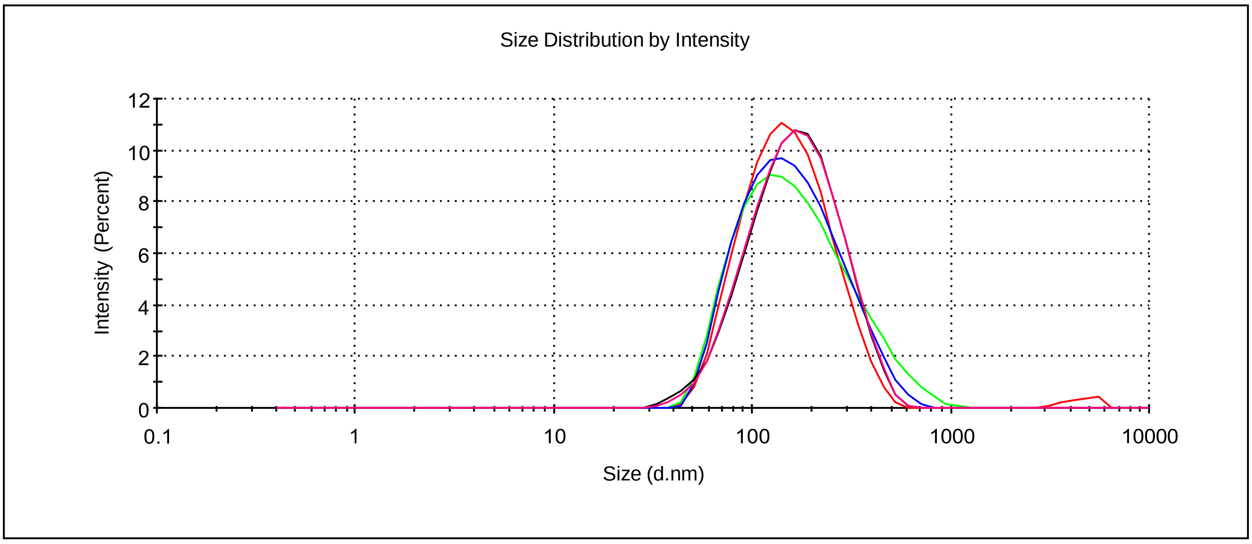

Figure S26. Size distribution of 5,11,17,23-tetra-*tert*-butyl-25,26,27,28-tetrakis[(N-(3′,3′-dimethyl- 3′-{3″-sulfonatopropyl})ammoniumpropyl)-carbamoylmethoxy]-2,8,14,20-tetrathiacalix[4]arene(*partial cone*) **(4)** in the presence of Ag^{I} in water ($1 \cdot \times 10^{-4}$ M), (1:1).

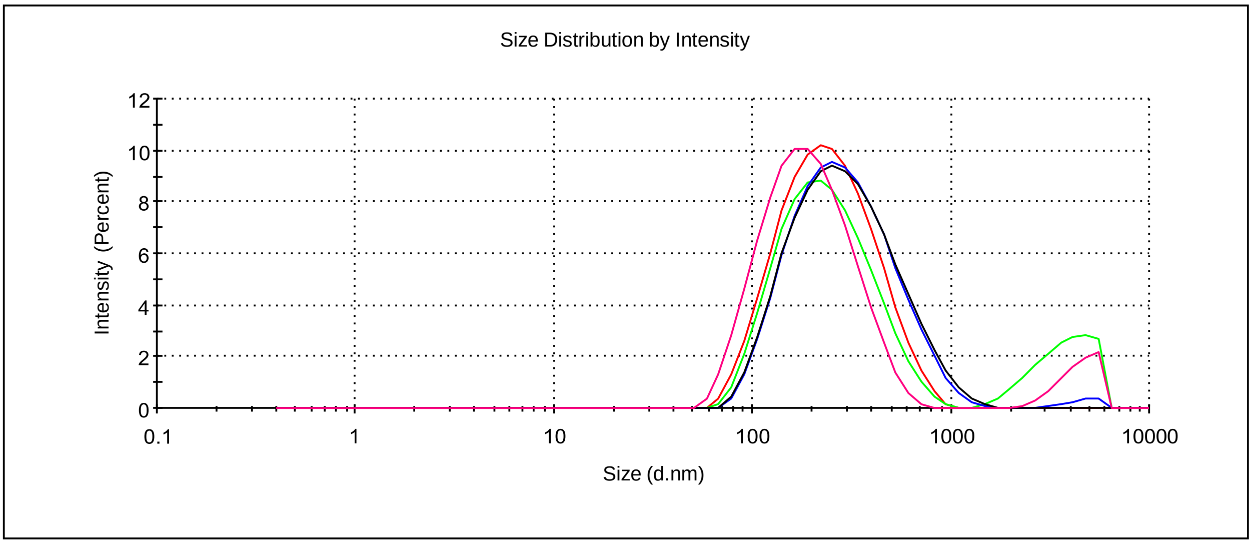

Figure S27. Size distribution of 5,11,17,23-tetra-*tert*-butyl-25,26,27,28-tetrakis[(N-(3′,3′-dimethyl- 3′-{3″-sulfonatopropyl})ammoniumpropyl)-carbamoylmethoxy]-2,8,14,20-tetrathiacalix[4]arene(*partial cone*) **(4)** in the presence of Ag^{I} in water ($1\cdot\times 10^{-5}$ M), (1:1).

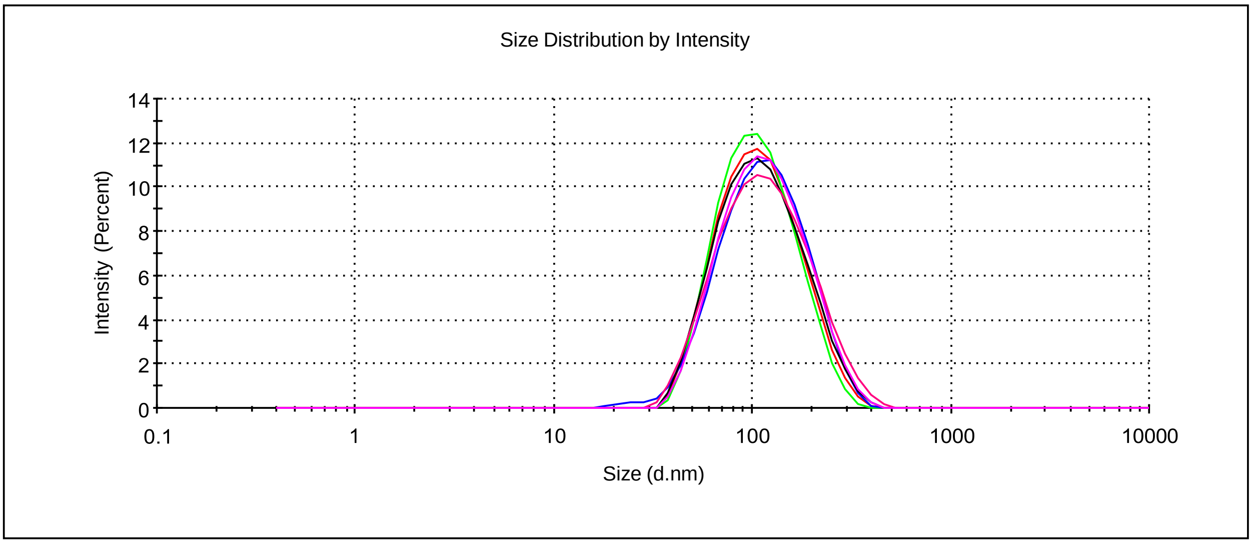

Figure S28. Size distribution of 5,11,17,23-tetra-*tert*-butyl-25,26,27,28-tetrakis[(N-(3′,3′-dimethyl- 3′-{3″-sulfonatopropyl})ammoniumpropyl)-carbamoylmethoxy]-2,8,14,20-tetrathiacalix[4]arene(*partial cone*) **(4)** in the presence of Ag^{I} in water ($1\cdot\times 10^{-4}$ M), (1:4).

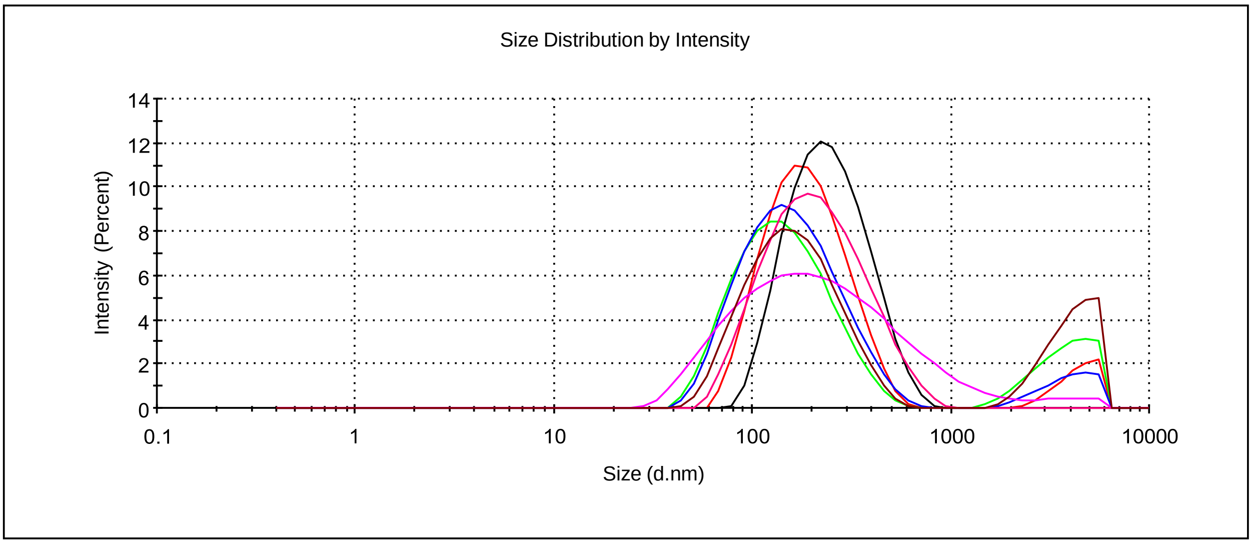

Figure S29. Size distribution of 5,11,17,23-tetra-*tert*-butyl-25,26,27,28-tetrakis[(N-(3′,3′-dimethyl- 3′-{3″-sulfonatopropyl})ammoniumpropyl)-carbamoylmethoxy]-2,8,14,20-tetrathiacalix[4]arene(*partial cone*) **(4)** in the presence of Ag^{I} in water (1·× 10^{-5} M), (1:4).

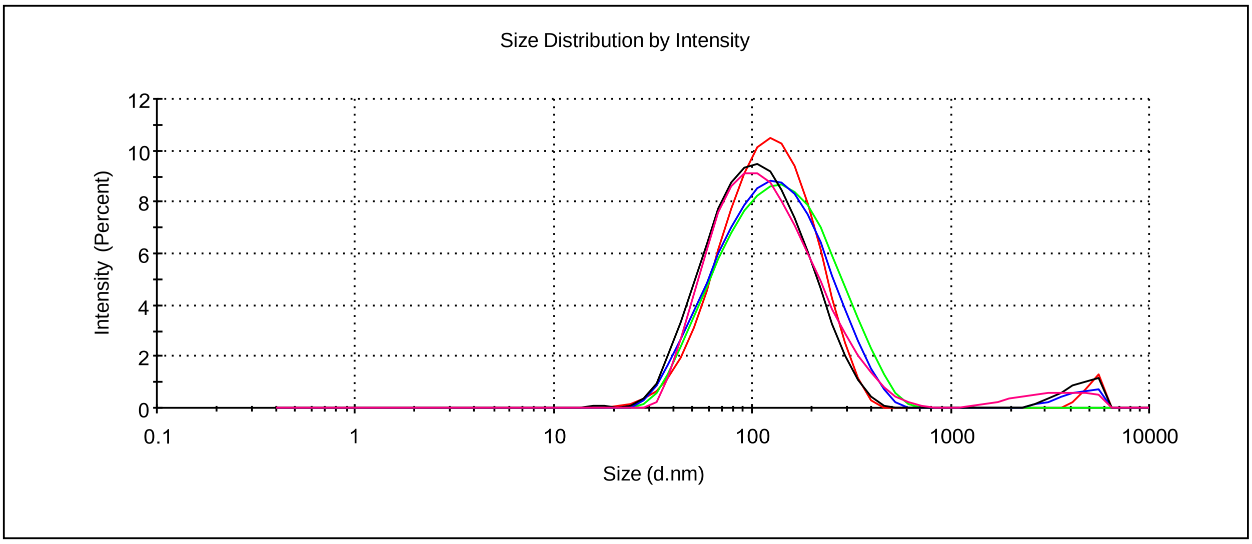

Figure S30. Size distribution of 5,11,17,23-tetra-*tert*-butyl-25,26,27,28-tetrakis[(N-(3′,3′-dimethyl- 3′-{3″-sulfonatopropyl})ammoniumpropyl)-carbamoylmethoxy]-2,8,14,20-tetrathiacalix[4]arene(*partial cone*) **(4)** in the presence of Ag^{I} in water (1·× 10^{-4} M), (1:10).

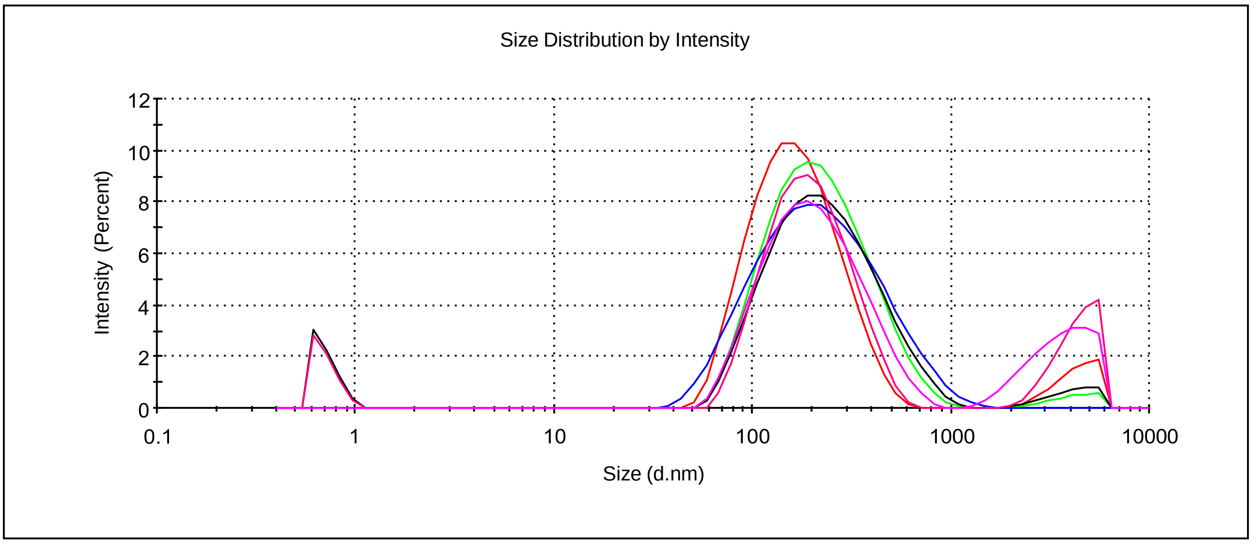

Figure S31. Size distribution of 5,11,17,23-tetra-*tert*-butyl-25,26,27,28-tetrakis[(N-(3′,3′-dimethyl- 3′-{3″-sulfonatopropyl})ammoniumpropyl)-carbamoylmethoxy]-2,8,14,20-tetrathiacalix[4]arene(*partial cone*) **(4)** in the presence of Ag^{I} in water ($1\cdot\times 10^{-5}$ M), (1:10).

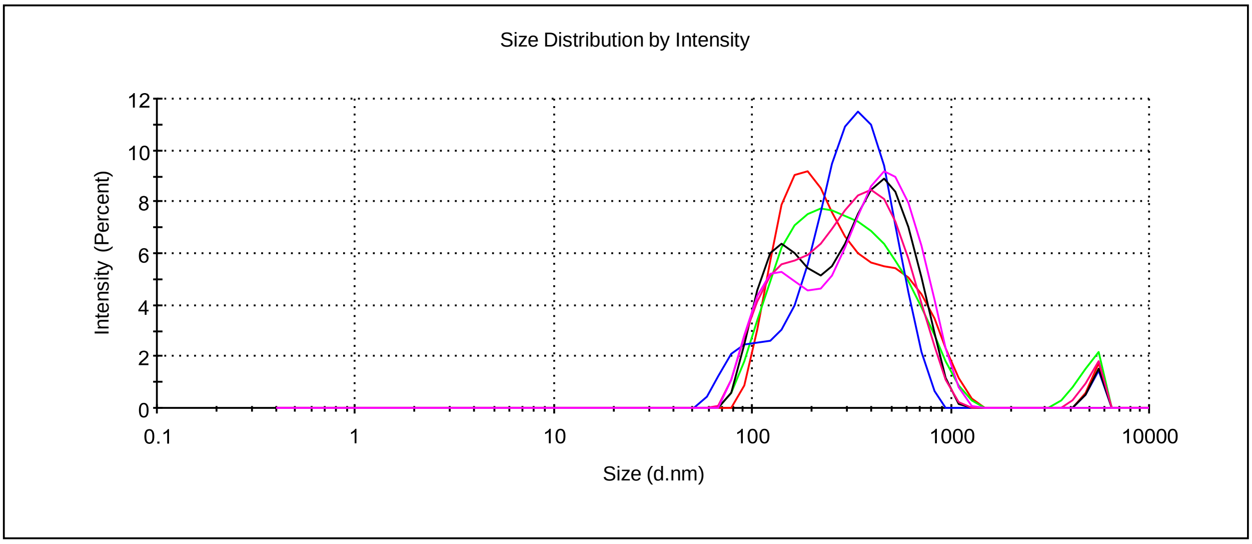

Figure S32. Size distribution of 5,11,17,23-tetra-*tert*-butyl-25,26,27,28-tetrakis[(N-(3′,3′-dimethyl- 3′-{3″-sulfonatopropyl})ammoniumpropyl)-carbamoylmethoxy]-2,8,14,20-tetrathiacalix[4]arene(*1,3-alternate*) **(5)** in water ($1\cdot\times 10^{-4}$ M).

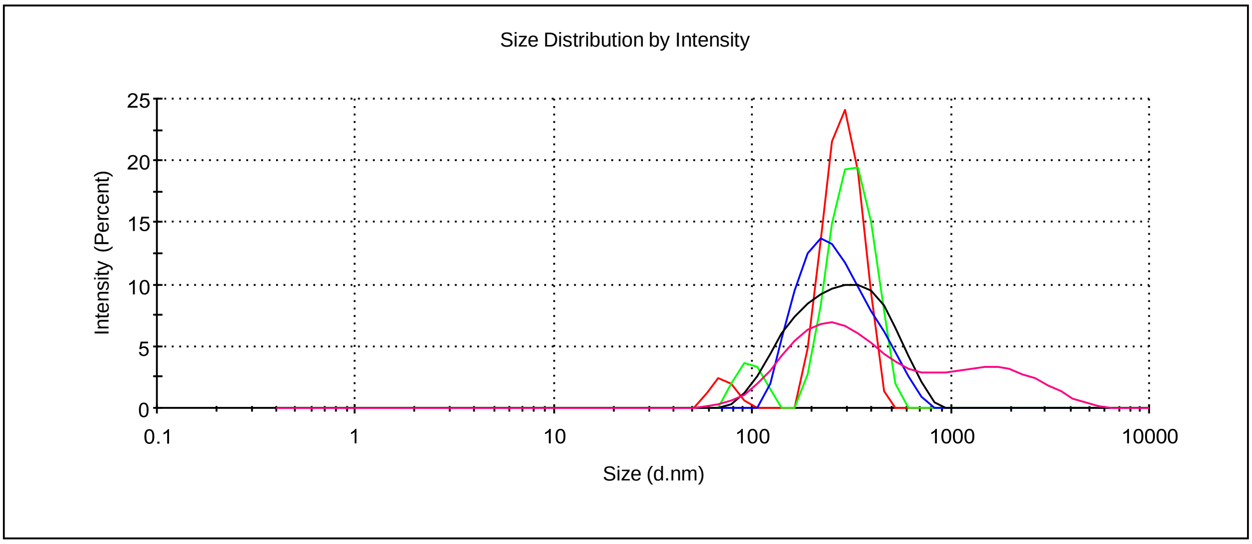

Figure S33. Size distribution of 5,11,17,23-tetra-*tert*-butyl-25,26,27,28-tetrakis[(N-(3′,3′-dimethyl- 3′-{3″-sulfonatopropyl})ammoniumpropyl)-carbamoylmethoxy]-2,8,14,20-tetrathiacalix[4]arene(*1,3-alternate*) **(5)** in water (1·× 10^{-5} M).

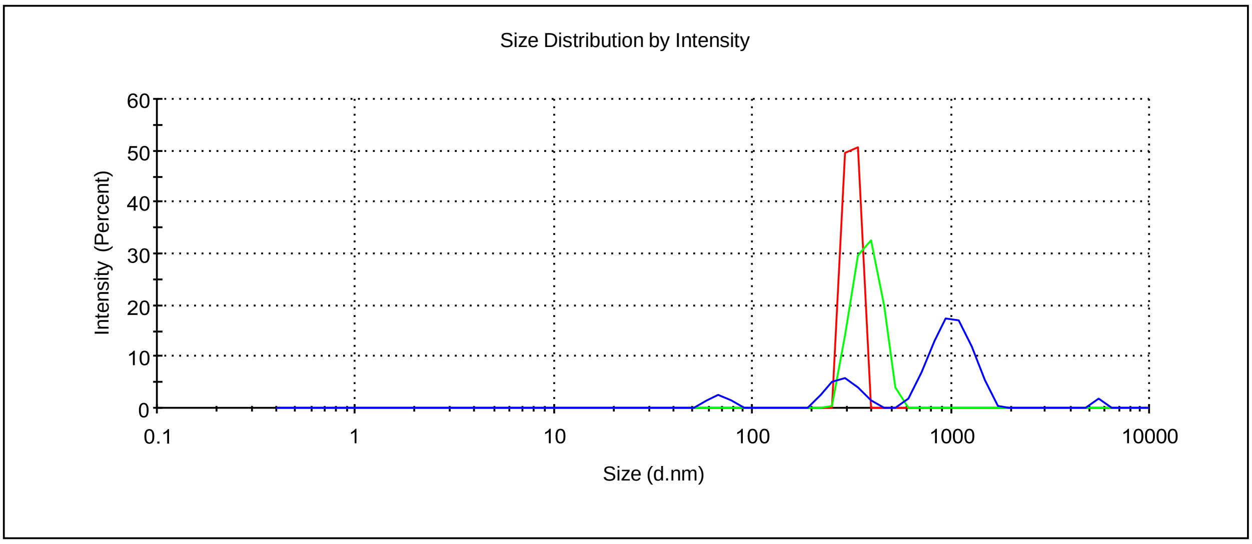

Figure S34. Size distribution of 5,11,17,23-tetra-*tert*-butyl-25,26,27,28-tetrakis[(N-(3′,3′-dimethyl- 3′-{3″-sulfonatopropyl})ammoniumpropyl)-carbamoylmethoxy]-2,8,14,20-tetrathiacalix[4]arene(*1,3-alternate*) **(5)** in water (1·× 10^{-6} M).

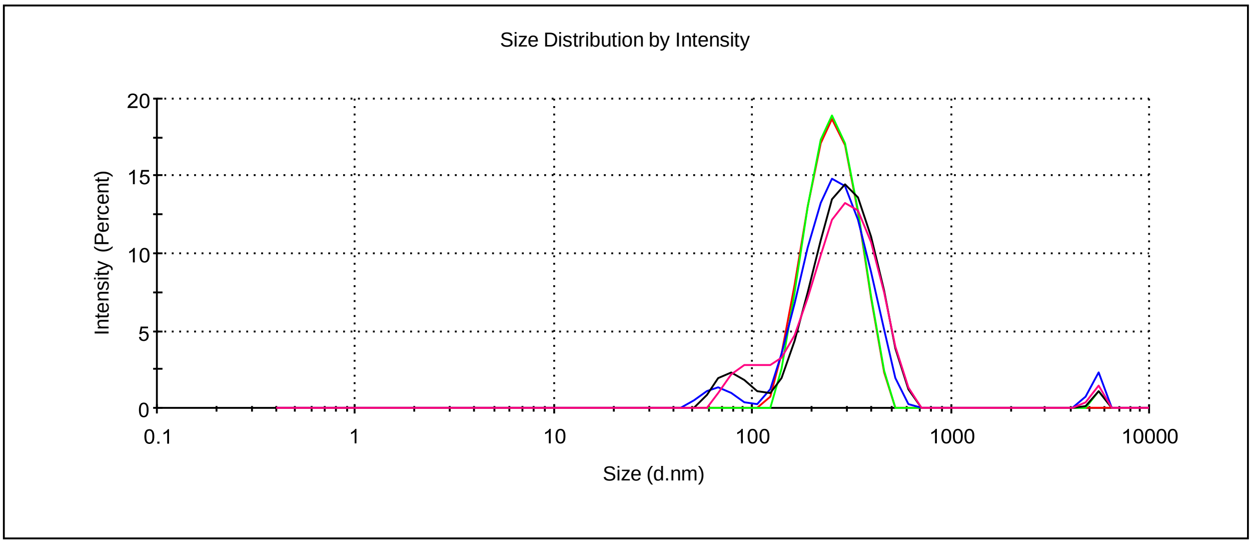

Figure S35. Size distribution of 5,11,17,23-tetra-*tert*-butyl-25,26,27,28-tetrakis[(N-(3′,3′-dimethyl- 3′-{3″-sulfonatopropyl})ammoniumpropyl)-carbamoylmethoxy]-2,8,14,20-tetrathiacalix[4]arene(*1,3-alternate*) **(5)** in the presence of Ag^I in water ($1\cdot\times 10^{-4}$ M), (1:1).

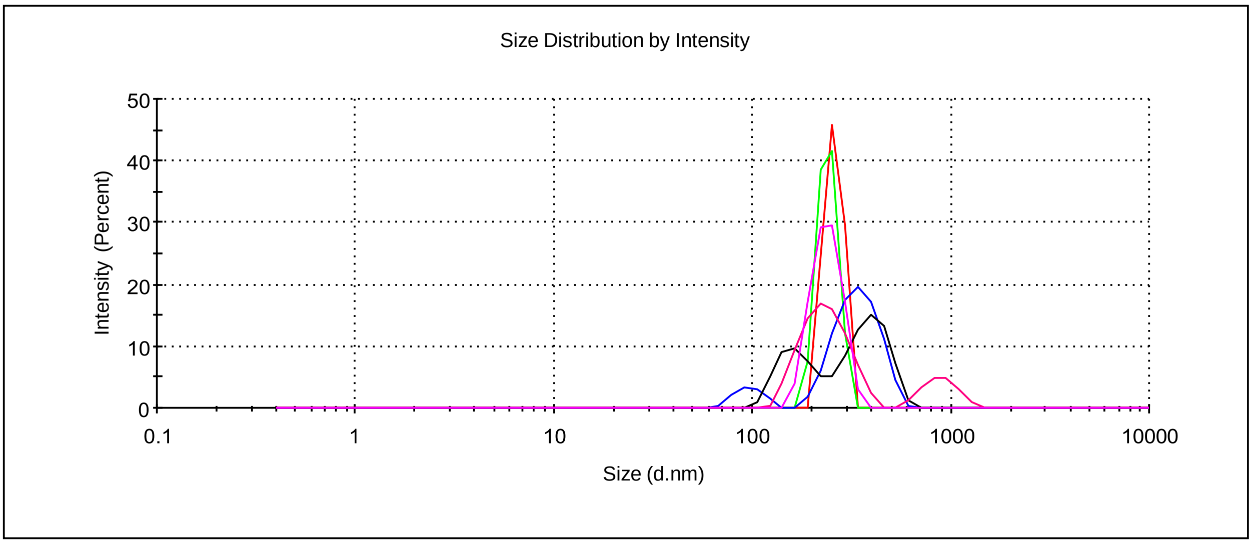

Figure S36. Size distribution of 5,11,17,23-tetra-*tert*-butyl-25,26,27,28-tetrakis[(N-(3′,3′-dimethyl- 3′-{3″-sulfonatopropyl})ammoniumpropyl)-carbamoylmethoxy]-2,8,14,20-tetrathiacalix[4]arene(*1,3-alternate*) **(5)** in the presence of Ag^I in water ($1\cdot\times 10^{-5}$ M), (1:1).

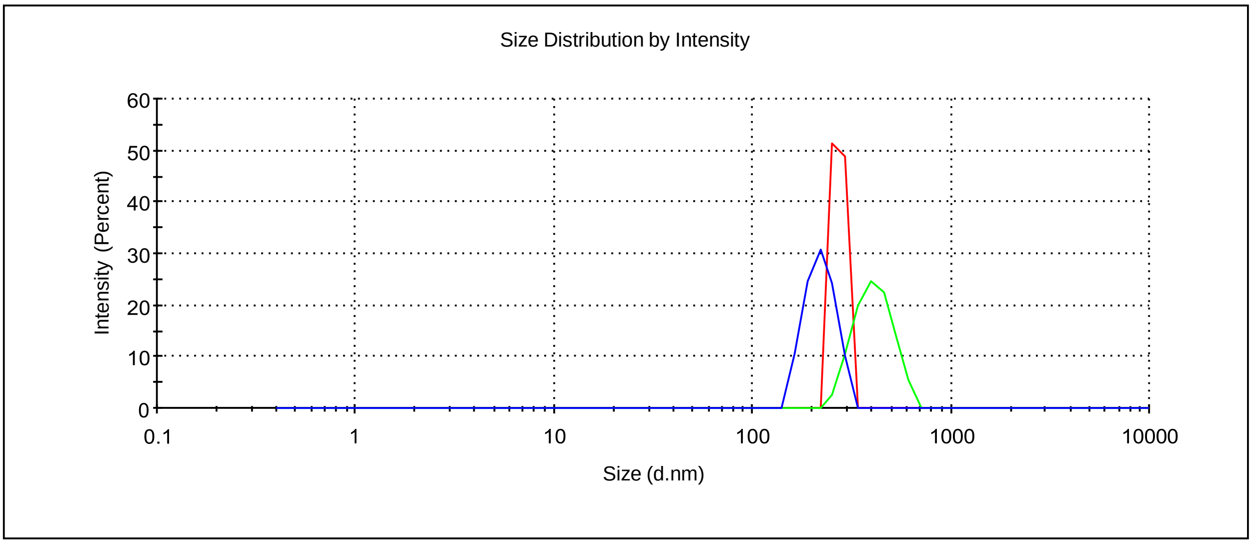

Figure S37. Size distribution of 5,11,17,23-tetra-*tert*-butyl-25,26,27,28-tetrakis[(N-(3′,3′-dimethyl- 3′-{3″-sulfonatopropyl})ammoniumpropyl)-carbamoylmethoxy]-2,8,14,20-tetrathiacalix[4]arene(*1,3-alternate*) **(5)** in the presence of Ag^{I} in water ($1\cdot\times 10^{-6}$ M), (1:1).

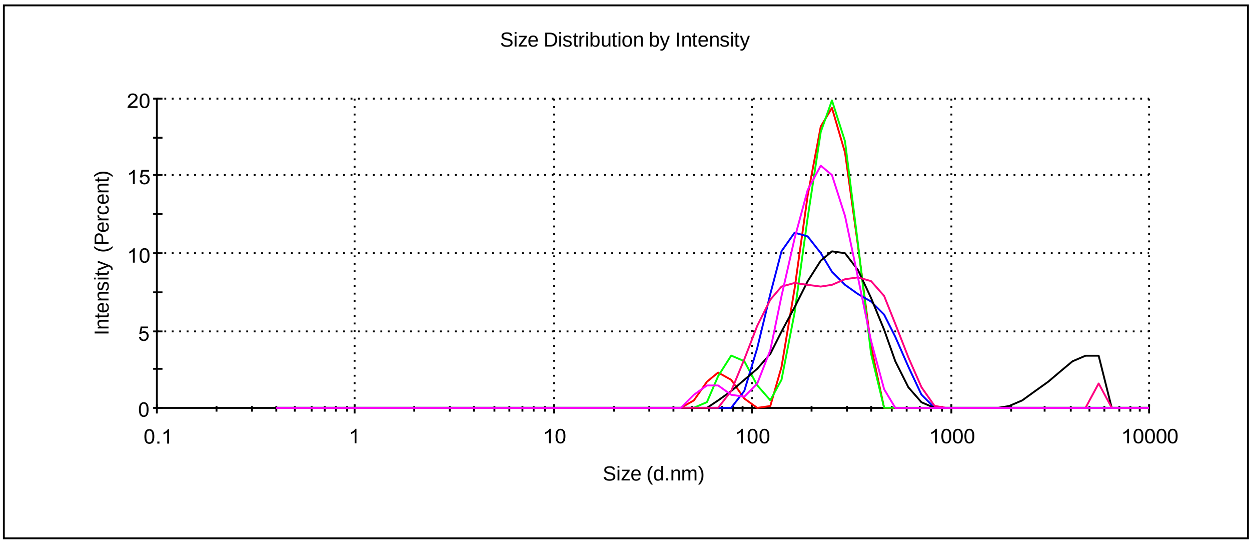

Figure S38. Size distribution of 5,11,17,23-tetra-*tert*-butyl-25,26,27,28-tetrakis[(N-(3′,3′-dimethyl- 3′-{3″-sulfonatopropyl})ammoniumpropyl)-carbamoylmethoxy]-2,8,14,20-tetrathiacalix[4]arene(*1,3-alternate*) **(5)** in the presence of Ag^{I} in water ($1\cdot\times 10^{-4}$ M), (1:4).

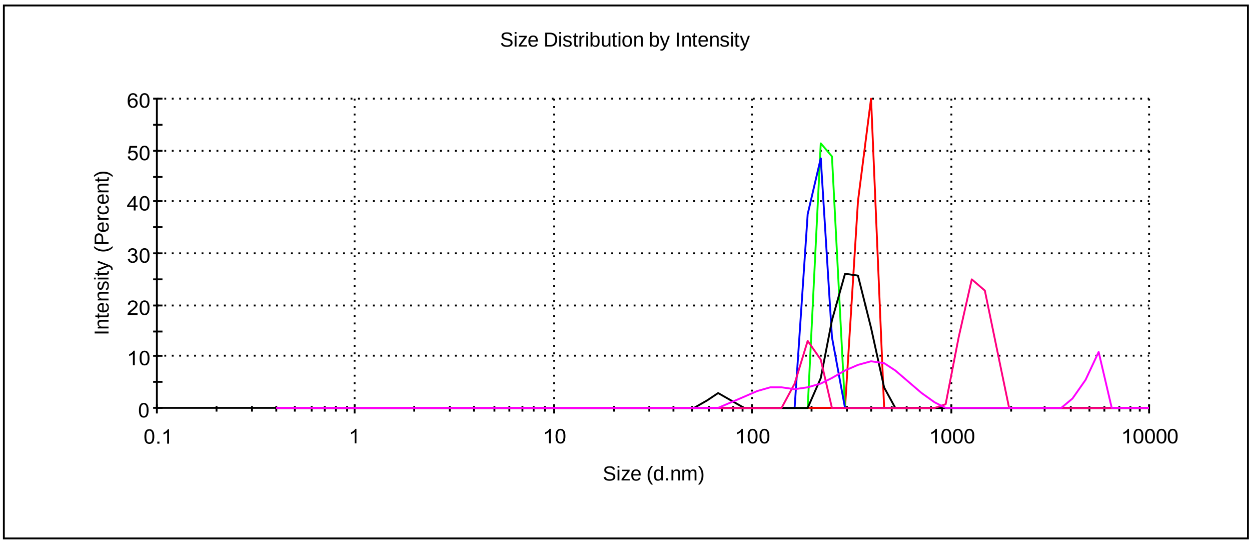

Figure S39. Size distribution of 5,11,17,23-tetra-*tert*-butyl-25,26,27,28-tetrakis[(N-(3′,3′-dimethyl-3′-{3″-sulfonatopropyl})ammoniumpropyl)-carbamoylmethoxy]-2,8,14,20-tetrathiacalix[4]arene(*1,3-alternate*) **(5)** in the presence of Ag^{I} in water ($1{\cdot}\times10^{-5}$ M), (1:4).

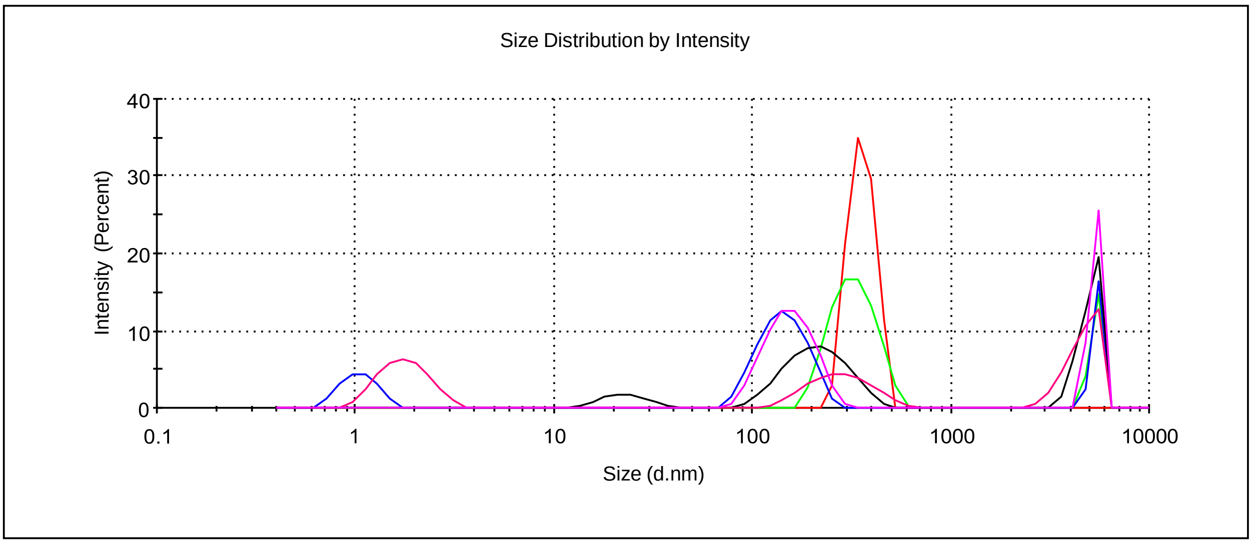

Figure S40. Size distribution of 5,11,17,23-tetra-*tert*-butyl-25,26,27,28-tetrakis[(N-(3′,3′-dimethyl-3′-{3″-sulfonatopropyl})ammoniumpropyl)-carbamoylmethoxy]-2,8,14,20-tetrathiacalix[4]arene(*1,3-alternate*) **(5)** in the presence of Ag^{I} in water ($1{\cdot}\times10^{-6}$ M), (1:4).

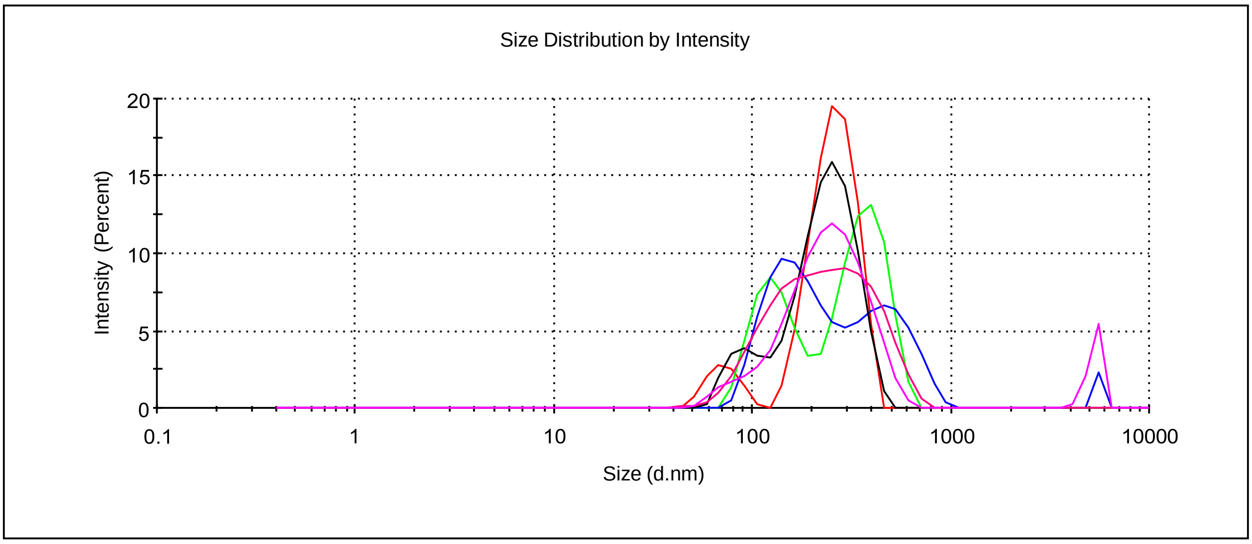

Figure S41. Size distribution of 5,11,17,23-tetra-*tert*-butyl-25,26,27,28-tetrakis[(N-(3′,3′-dimethyl- 3′-{3″-sulfonatopropyl})ammoniumpropyl)-carbamoylmethoxy]-2,8,14,20-tetrathiacalix[4]arene(*1,3-alternate*) **(5)** in the presence of Ag^{I} in water ($1{\cdot}\times10^{-4}$ M), (1:10).

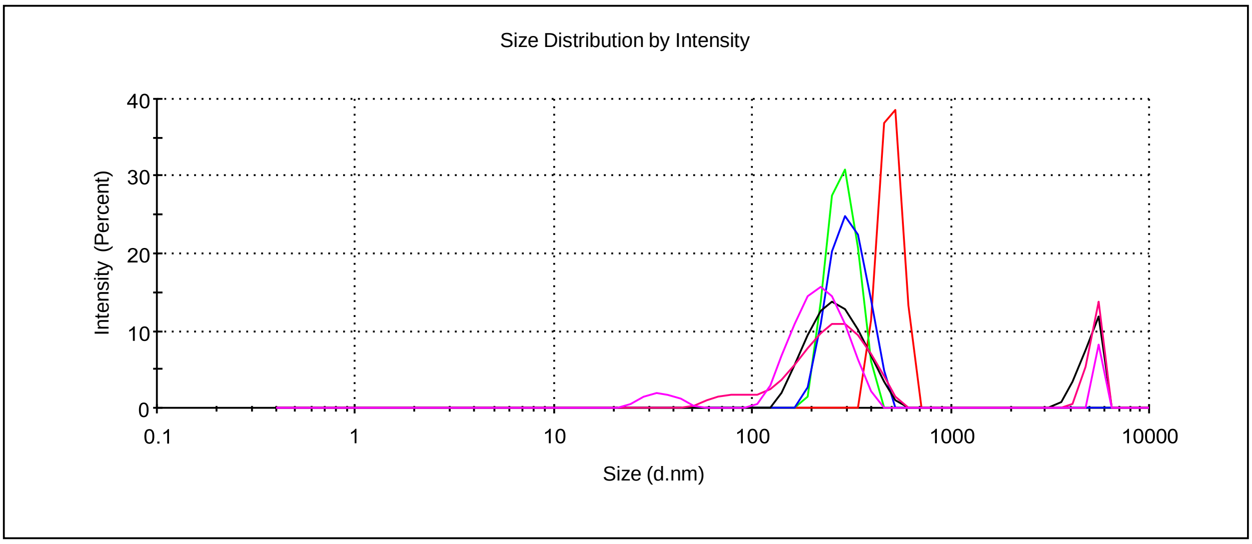

Figure S42. Size distribution of 5,11,17,23-tetra-*tert*-butyl-25,26,27,28-tetrakis[(N-(3′,3′-dimethyl- 3′-{3″-sulfonatopropyl})ammoniumpropyl)-carbamoylmethoxy]-2,8,14,20-tetrathiacalix[4]arene(*1,3-alternate*) **(5)** in the presence of Ag^{I} in water ($1{\cdot}\times10^{-5}$ M), (1:10).

3. UV spectra

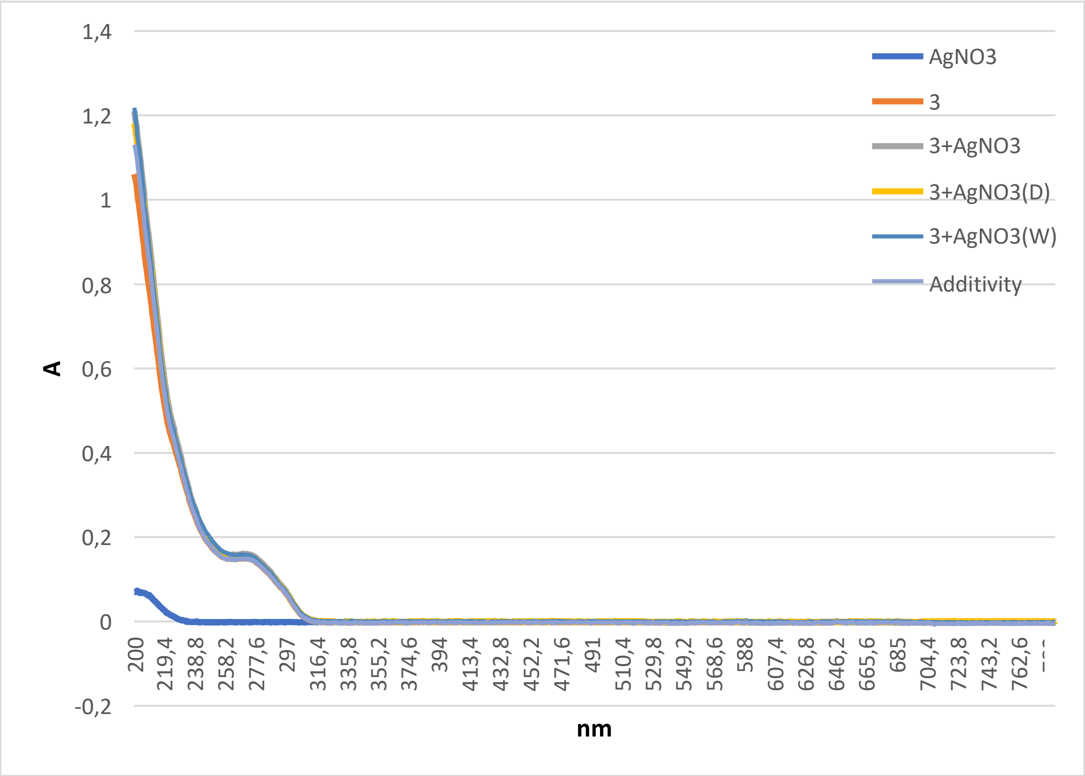

Figure S43. UV-*vis* spectra of $AgNO_3$ in the presence of **3** ($1 \cdot \times 10^{-5}$ M) ,(UV spectra of the solutions were recorded on the day of their preparation, after 1 (D) and after 7 days (W)).

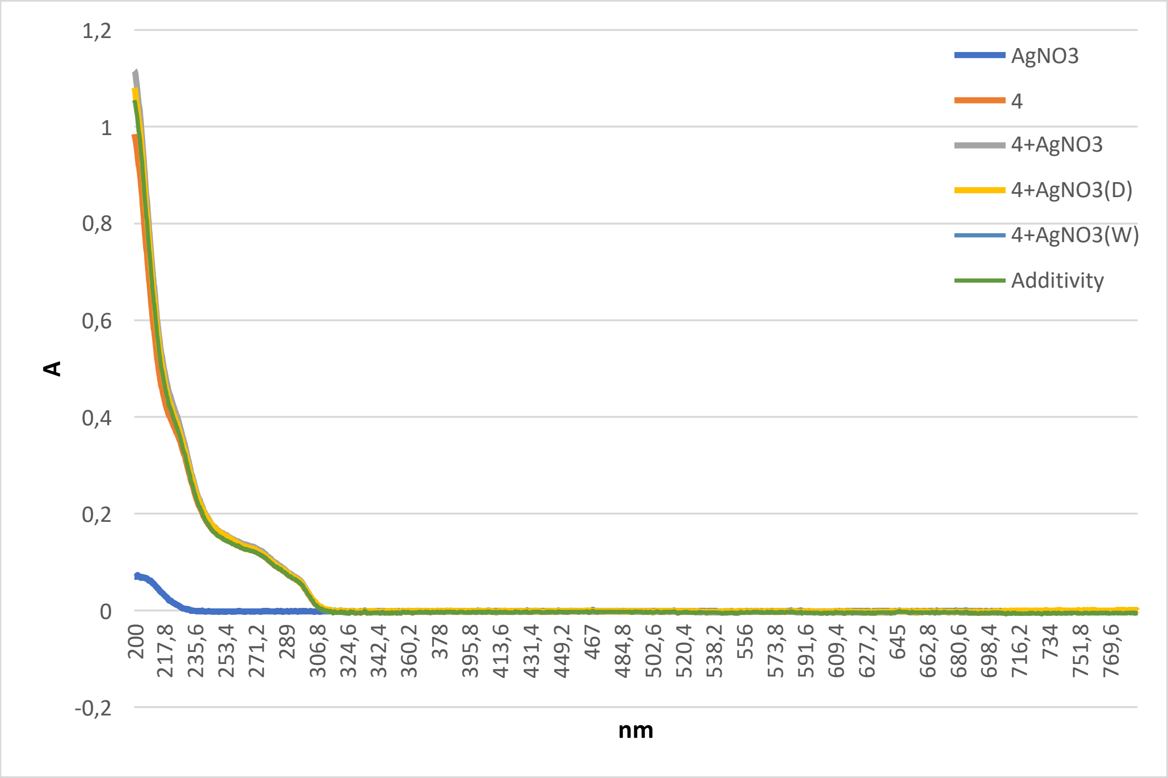

Figure S44. UV-*vis* spectra of $AgNO_3$ in the presence of **4** ($1 \cdot \times 10^{-5}$ M), (UV spectra of the solutions were recorded on the day of their preparation, after 1 (D) and after 7 days (W)).

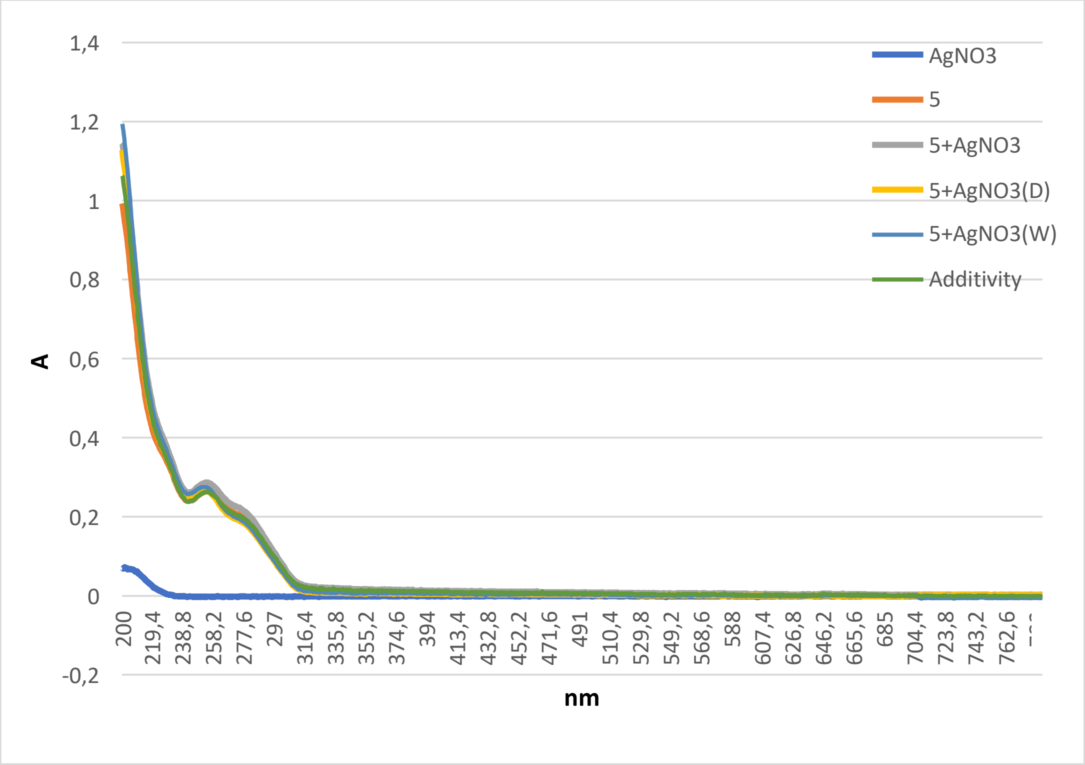

Figure S45. UV-*vis* spectra of $AgNO_3$ in the presence of **5** (1·× 10^{-5} M), (UV spectra of the solutions were recorded on the day of their preparation, after 1 (D) and after 7 days (W)).

4. NMR spectra of associates.

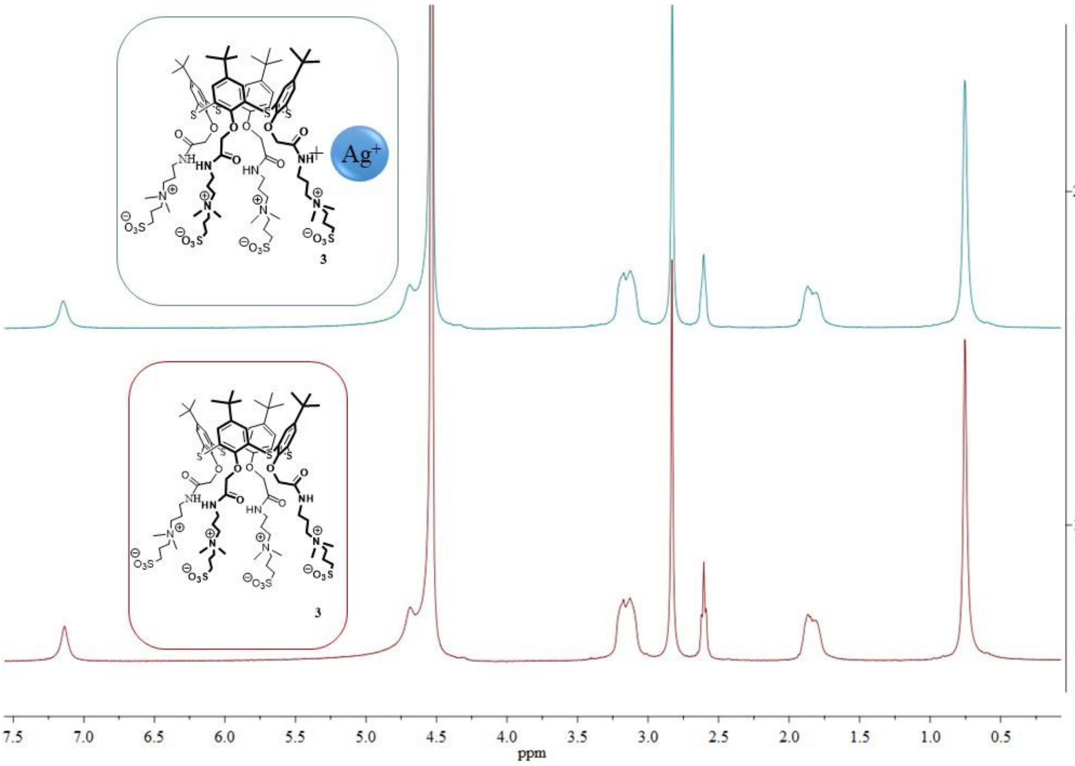

Figure S46. ^{1}H NMR spectra of macrocycle **3** ($1 \cdot \times 10^{-3}$ M) and associates **3**/Ag^{+} at a ratio of 1:4 inD_2O at 298 K.

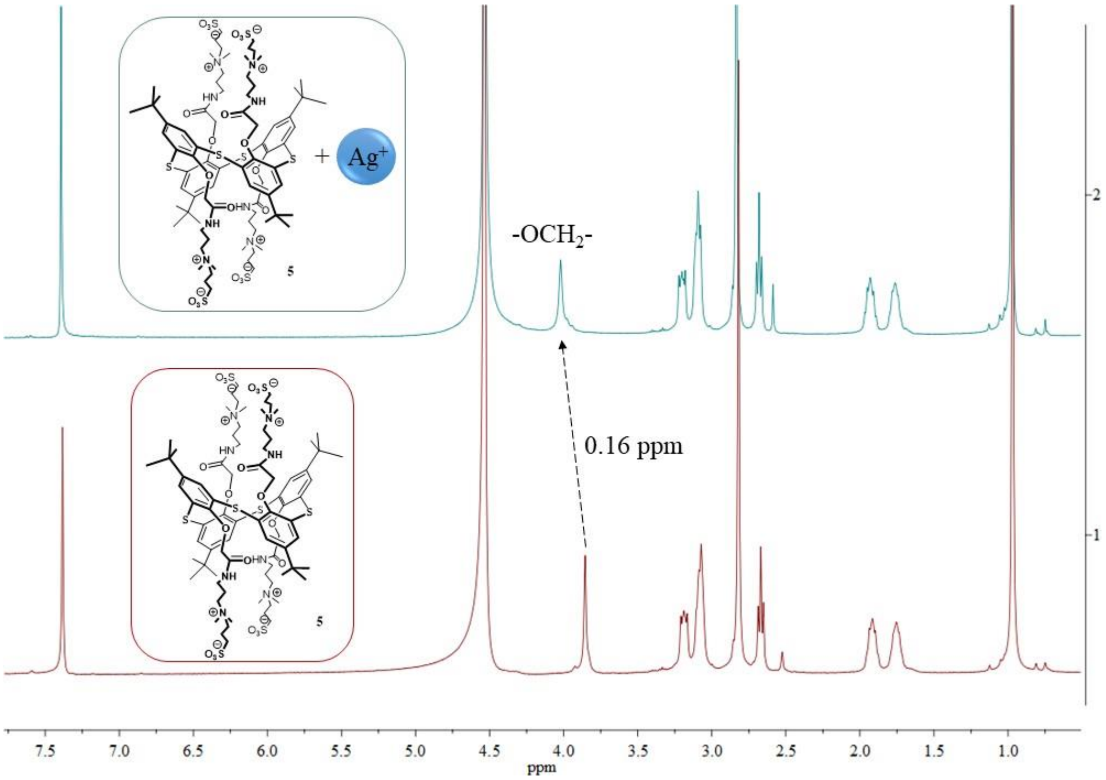

Figure S47. ^{1}H NMR spectra of macrocycle **5** ($1 \cdot \times 10^{-3}$ M) and associates **5**/Ag^{+} at a ratio of 1:4 inD_2O at 298 K.

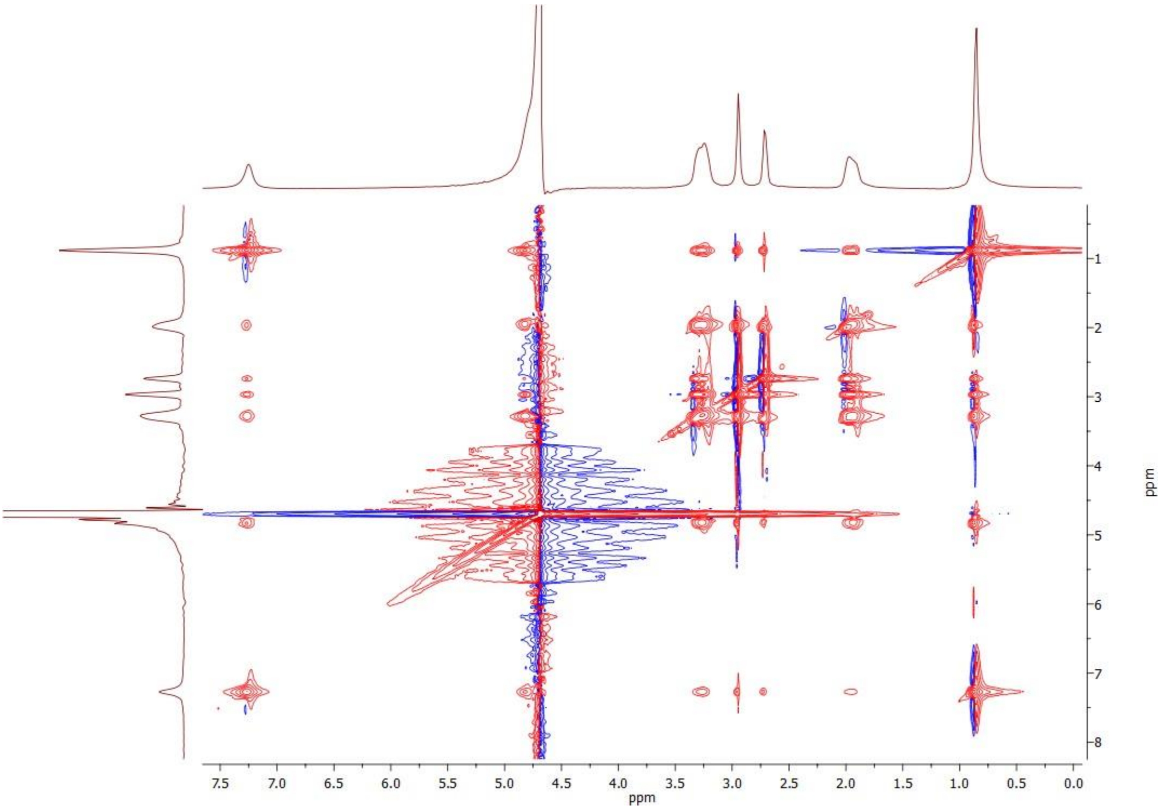

Figure S48. 2D ^{1}H–^{1}H NOESY NMR spectra of macrocycle **3** (1·× 10^{-3} M) in D_2O at 298 K.

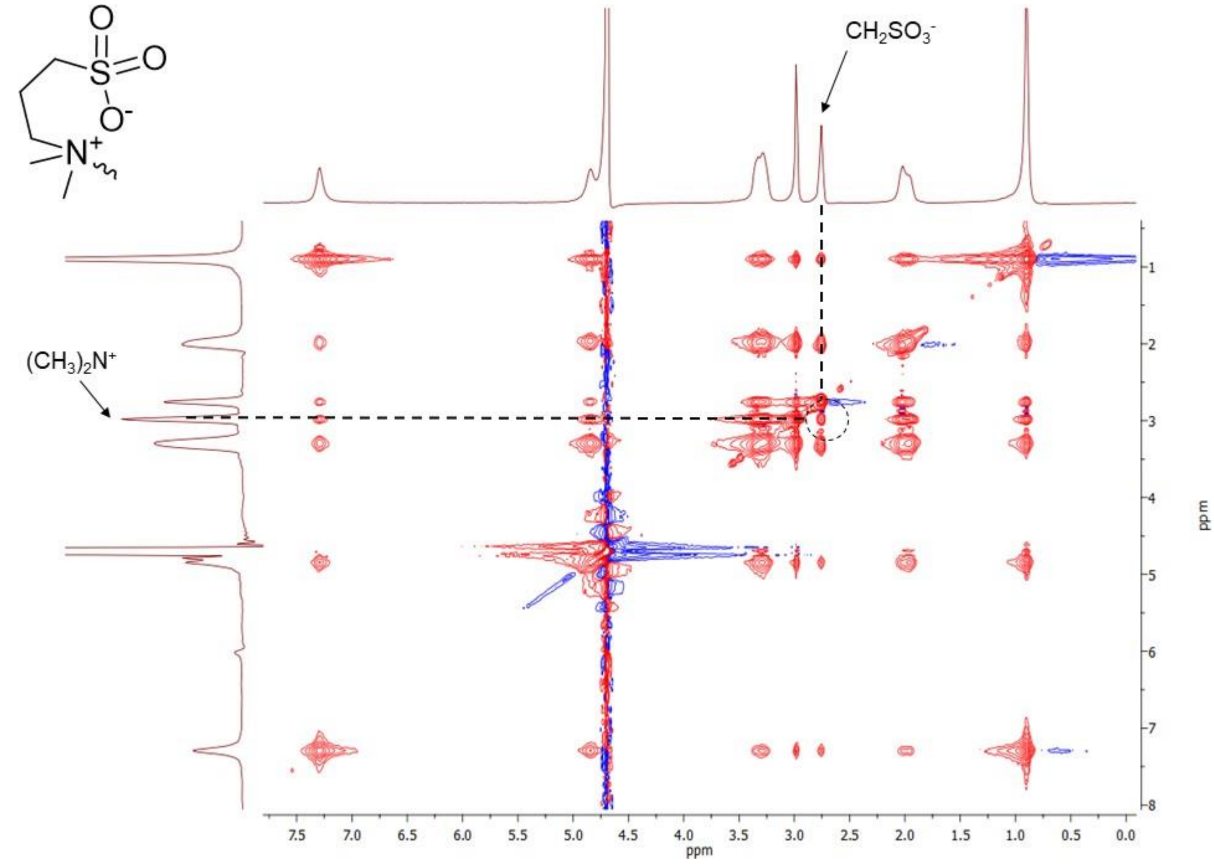

Figure S49. 2D ^{1}H–^{1}H NOESY NMR spectra of associates **3**/Ag^{+} at a ratio of 1:4 (1·× 10^{-3} M) inD$_2$O at 298 K.

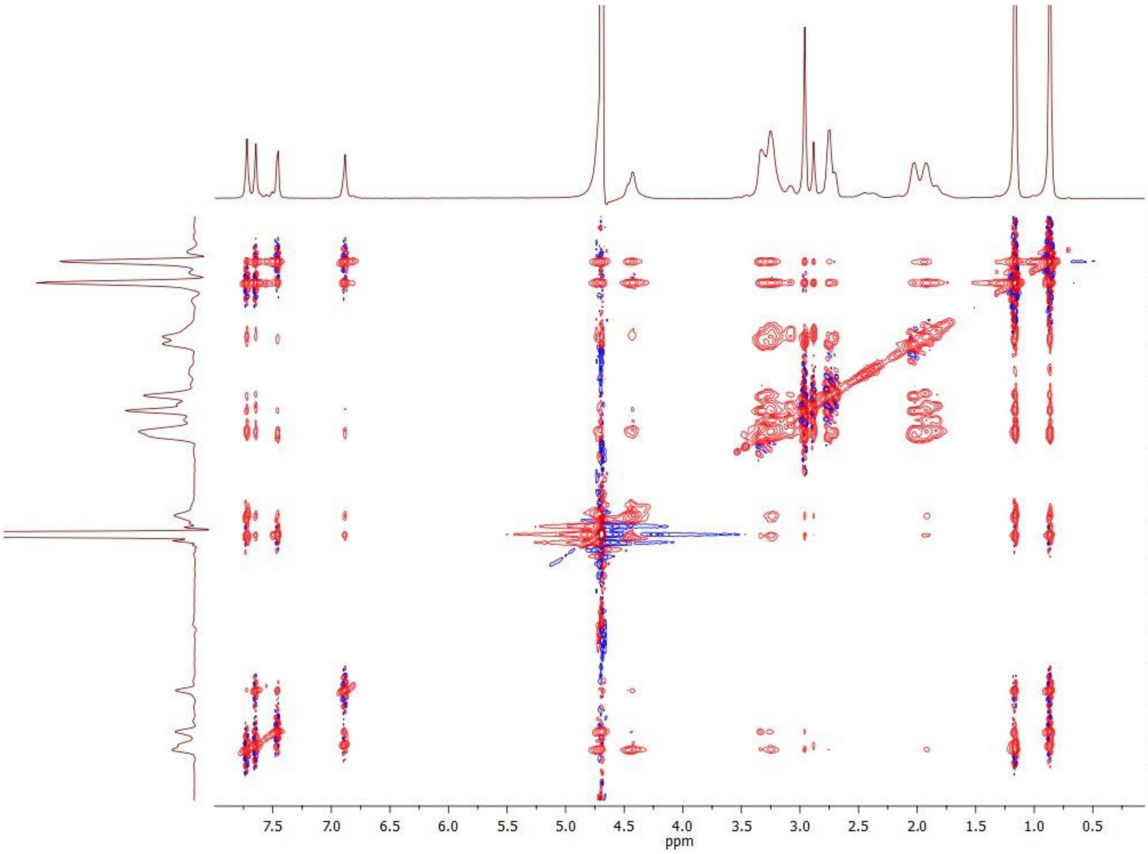

Figure S50. 2D ^{1}H–^{1}H NOESY NMR spectra of macrocycle **4** ($1 \cdot\times 10^{-3}$ M) in D_2O at 298 K.

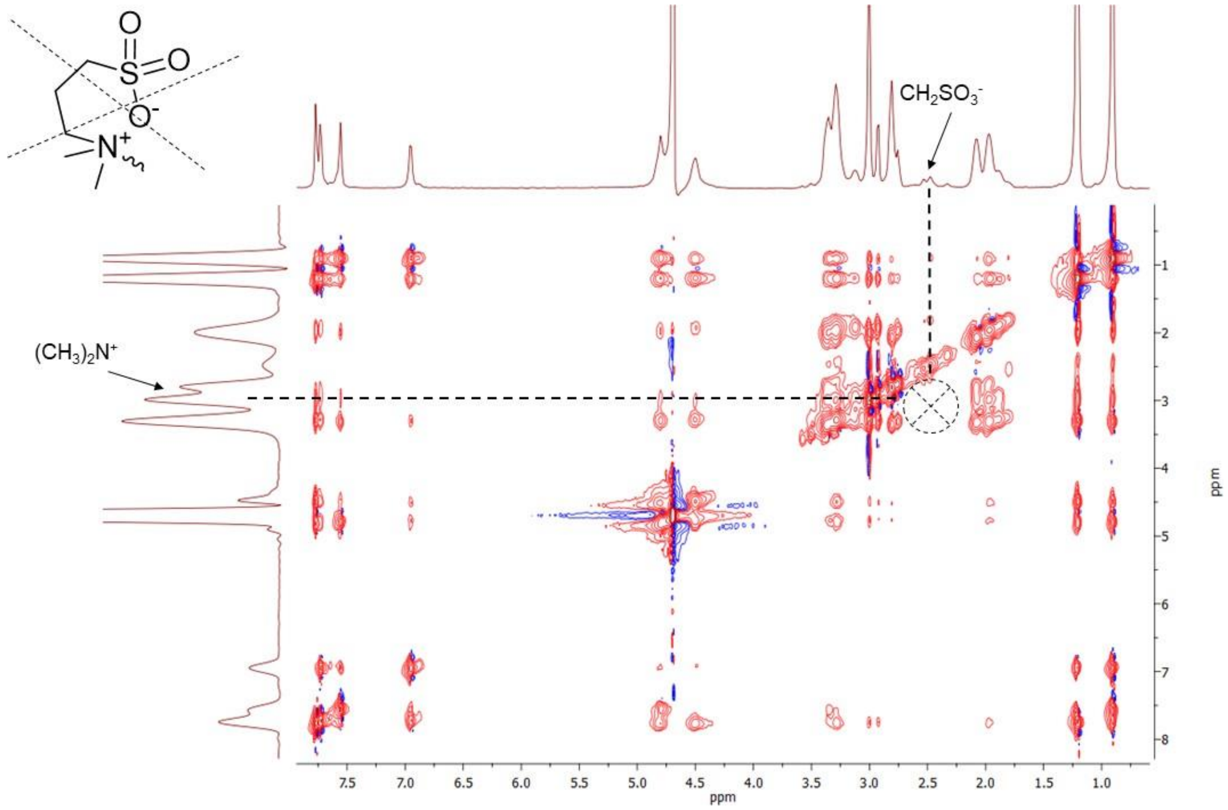

Figure S51. 2D ^{1}H–^{1}H NOESY NMR spectra of associates **4**/Ag^{+} at a ratio of 1:4 ($1 \cdot\times 10^{-3}$ M) inD$_2$O at 298 K.

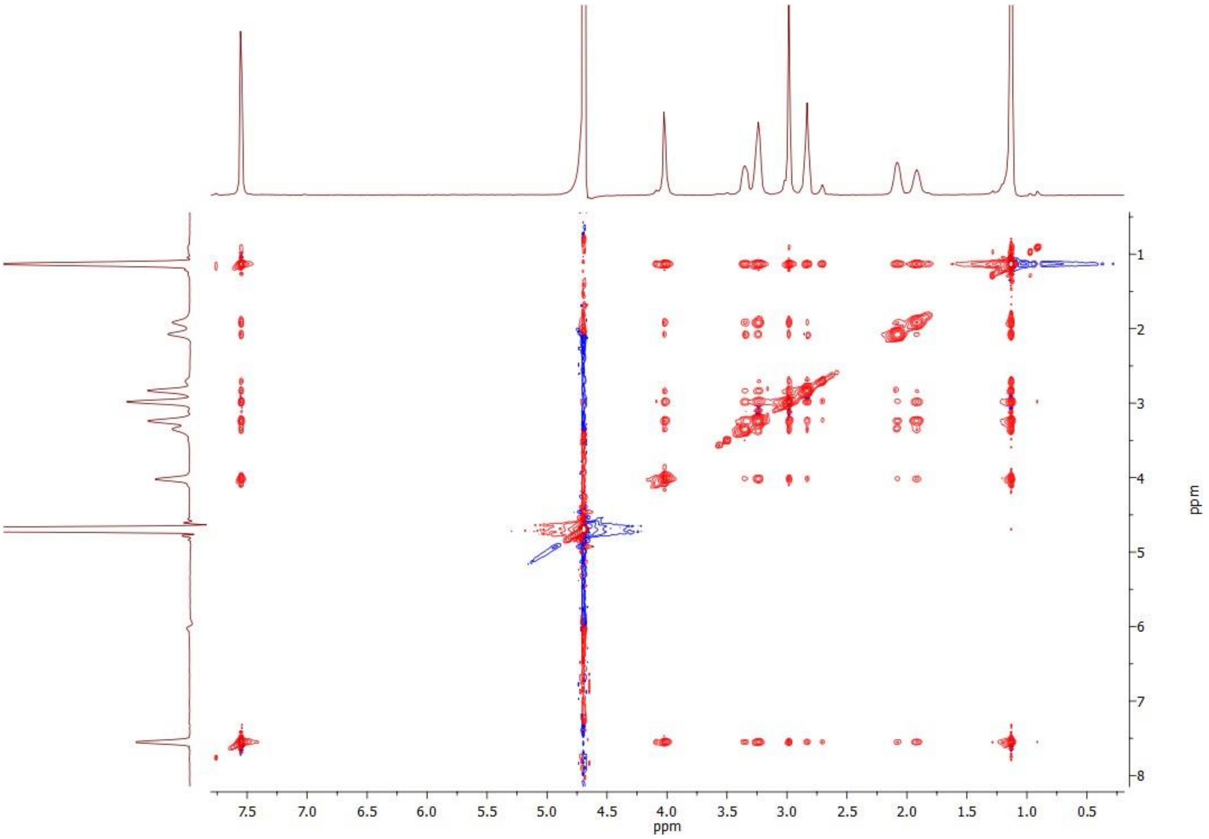

Figure S52. 2D ^{1}H–^{1}H NOESY NMR spectra of macrocycle **5** (1·× 10^{-3} M) in D_2O at 298 K.

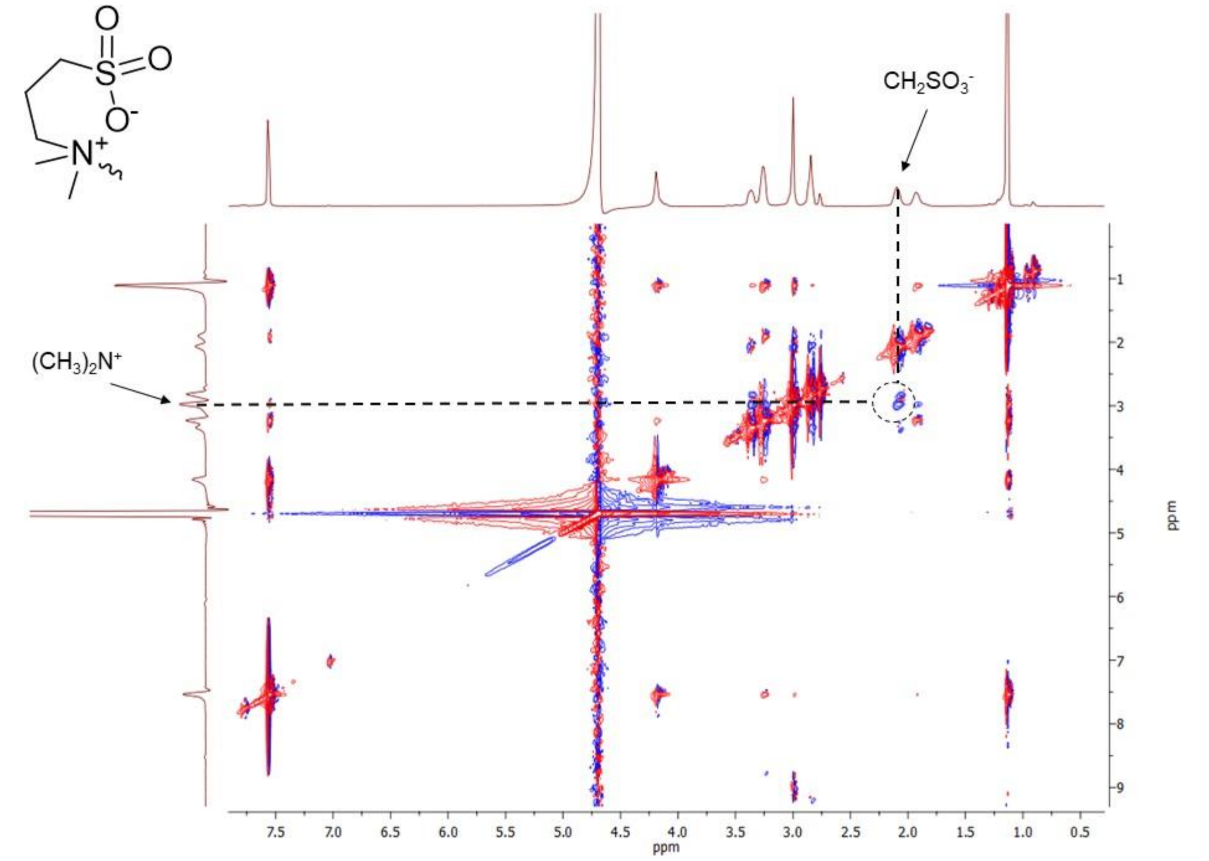

Figure S53. 2D ^{1}H–^{1}H NOESY NMR spectra of associates **5**/Ag^+ at a ratio of 1:4 (1·× 10^{-3} M) inD2O at 298 K.

5. Tables

Table S1. Size of aggregates formed during association of compound **3** in water in the presence / absence of Ag (I) (hydrodynamic diameters d, nm and PDI).

c, mol/L	**3**		**3+Ag⁺** 1:1		**3+Ag⁺** 1:4		**3+Ag⁺** 1:10	
	PDI	d, nm	PDI	d, nm	PDI	d, nm	PDI	d, nm
$1\cdot\times 10^{-4}$	0.82±0.15	421±74	**0.17±0.02**	**168±7**	**0.18±0.01**	**243±13**	0.30±0.05	817±48
$1\cdot\times 10^{-5}$	0.54±0.17	335±47	0.31±0.06	152±27	0.27±0.03	756±93	0.75±0.16	446±92
$1\cdot\times 10^{-6}$	0.48±0.33	317±203	0.52±0.12	193±32	0.35±0.09	234±50	-	-

Table S2. Size of aggregates formed during association of compound **4** in water in the presence / absence of Ag (I) (hydrodynamic diameters d, nm and PDI).

c, mol/L	**4**		**4+Ag⁺** 1:1		**4+Ag⁺** 1:4		**4+Ag⁺** 1:10	
	PDI	d, nm	PDI	d, nm	PDI	d, nm	PDI	d, nm
$1\cdot\times 10^{-4}$	0.38±0.12	201±32	**0.21±0.01**	**182±11**	**0.19±0.01**	**123±6**	**0.27±0.02**	**147±7**
$1\cdot\times 10^{-5}$	0.31±0.15	143±96	0.34±0.11	285±60	0.35±0.11	249±58	0.36±0.06	235±37
$1\cdot\times 10^{-6}$	-	-	-	-	-	-	-	-

Table S3. Size of aggregates formed during association of compound **5** in water in the presence / absence of Ag (I) (hydrodynamic diameters d, nm and PDI).

c, mol/L	**5**		**5+Ag⁺** 1:1		**5+Ag⁺** 1:4		**5+Ag⁺** 1:10	
	PDI	d, nm	PDI	d, nm	PDI	d, nm	PDI	d, nm
$1\cdot\times 10^{-4}$	0.38±0.02	381±65	0.35±0.05	298±13	0.45±0.04	274±50	0.52±0.04	260±62
$1\cdot\times 10^{-5}$	0.52±0.09	289±46	0.60±0.08	284±62	0.86±0.21	306±78	0.70±0.17	273±31
$1\cdot\times 10^{-6}$	0.91±0.13	351±45	0.97±0.05	249±36	0.82±0.12	243±97	-	-

Table S4. Size of aggregates formed during association of compounds **3** and **4** in water in the presence / absence of Ag (I) (hydrodynamic diameters d, nm and PDI).

c, mol/L	**3**		**$3+Ag^+$** 1:4		**4**		**$4+Ag^+$** 1:4	
	PDI	d, nm	PDI	d, nm	PDI	d, nm	PDI	d, nm
$1\cdot\times 10^{-4}$	0.82±0.15	421±74	**0.18±0.01**	**243±13**	0.38±0.12	0.38±0.12	**0.19±0.01**	**123±6**
$1\cdot\times 10^{-5}$	0.54±0.17	335±47	0.27±0.03	756±93	0.31±0.15	0.31±0.15	0.35±0.11	249±58
$1\cdot\times 10^{-6}$	0.48±0.33	317±203	0.35±0.09	234±50	-	-	-	-
